# Multi-timescale optimization scheduling of integrated energy systems oriented towards generalized energy storage services

**DOI:** 10.1038/s41598-025-92601-9

**Published:** 2025-03-12

**Authors:** Yunshou Mao, Zhihong Cai, Xianan Jiao, Dafeng Long

**Affiliations:** 1 School of Electronic Information and Electrical Engineering, Huizhou University, Huizhou, 516007 Guangdong P.R. China; 2 Guangdong Provincial Key Laboratory of Electronic Functional Materials and Devices, Huizhou University, Huizhou, 516007 Guangdong P.R. China; 3 Electric Power Research Institute of EHV Power Transmission Company, CSG, Guangzhou, 510663 Guangdong P.R. China; 4 School of automation, Guangdong university of technology, Guangzhou, 510006 Guangdong P.R. China

**Keywords:** Generalized energy storage, Two-stage robust optimization, Column-and-constraint generation, Integrated energy system, Air conditioning virtual energy storage, Electrical and electronic engineering, Renewable energy

## Abstract

This paper addresses the limitations of existing research that focuses on single-sided resources and two-timescale optimization, overlooking the coordinated response of various energy storage resources across different timescales in comprehensive energy systems. To tackle these shortcomings, the study integrates flexible demand-side resources, such as electric vehicles (EVs), hydrogen storage, and air conditioning clusters, as generalized energy storage. It explores their impact on the operation cost of the comprehensive energy system across three stages: day-ahead, intraday, and real-time. The paper establishes an optimization scheduling model for mobile energy storage, hydrogen storage, and virtual energy storage of air conditioning clusters, considering the physical and temporal constraints of different storage devices, aiming to minimize the operational cost. The day-ahead stage employs C&CG to address the uncertainty of wind and photovoltaic power generations, while the intraday stage synergizes hydrogen storage, gas turbines, and demand-side substitutable and transferable loads to mitigate renewable energy fluctuations. The real-time stage leverages the virtual energy storage model of air conditioning clusters for rapid response to renewable energy deviations. Case studies validate the effectiveness of the model, demonstrating that multi-timescale optimization of generalized energy storage in comprehensive energy systems can significantly reduce operational costs and enhance system reliability.

**Supplementary Information:**

The online version contains supplementary material available at 10.1038/s41598-025-92601-9.

## Introduction

Nowadays, the pursuit of sustainable energy solutions has led to the emergence of integrated energy systems (IES) that leverage smart grid technologies to manage a diverse array of energy resources. These systems are designed to optimize energy utilization by integrating various energy forms, such as electricity, heat, and gas, within a given region^1,2^. The core objective of IES is to enhance the efficiency and reliability of energy supply while maximizing the use of renewable energy sources, such as wind and solar power. Despite the potential benefits, the operation of IES faces significant challenges due to the stochastic nature of renewable energy generation and the variability of load demands. The unpredictability of these factors limits the economic efficiency of IES, necessitating innovative solutions to enhance system stability and performance^3,4^.

In existing research, scholars have conducted extensive studies on the coordinated control and scheduling optimization of IES considering demand-side responses. The work^5^ established a flexible integrated demand response model for an energy system, leveraging price signals to motivate consumers to curtail or shift their energy usage. This model also incorporates various energy storage technologies to enhance the system’s ability to manage the dynamics of demand and supply effectively. However, the model does not consider the potential of EVs to act as energy storage units. In the face of uncertainties in IES operations, such as renewable energy variability and load forecasting errors, optimization strategies like stochastic, robust, and interval methods are widely applied to ensure system resilience and efficiency^6,7^. The work^8^ focused on the energy scheduling problem of multi-energy rural microgrids and used the Latin hypercube sampling and scenario reduction techniques to handle multiple uncertainties. The work^9^ focused on the electricity-hydrogen integrated energy systems, proposing a multi-stage scheduling framework to balance the economy, security, and computational burden of the system, thereby improving the system operation performance. However, the works^8,9^ fail to consider the collaborative application of multiple energy storage devices (including EVs), missing the opportunity to achieve greater economic and environmental benefits.

In the realm of multi-time-scale coordinated optimization of IES, significant attention has been received in recent research. The multi-time-scale scheduling strategy of the system mainly has two objectives. One is to make full use of energy storage devices to achieve the goal of peak-shaving and valley-filling, improving the system’s economy and overall efficiency. The other is to address the issue that the prediction accuracy of new energy output gradually decreases as the time scale increases. By adopting a multi-time-scale scheduling strategy, the uncertainty of the system can be better mitigated. To achieve these two goals, the existing scheduling methods can be mainly categorized into single-layer scheduling strategies and hierarchical scheduling strategies.

In single-layer scheduling methods, in terms of economy, a relatively large time scale is required in the optimization process. This is to cover the coupling and variation characteristics among multiple energy flows within the system as comprehensively as possible, so as to fully utilize the complementary characteristics of energy flows within the system and the functions of energy storage devices. Therefore, the sampling time scale is generally large, and the most commonly used scheduling scale currently is 1 h. For example, in reference^10^, a mixed-integer linear programming model was established with a 1-hour scheduling cycle, aiming to minimize the power purchase cost and equipment start-stop cost. Reference^11^ obtained hourly-level scheduling instructions for equipment by establishing chance constraints. Reference^12^ solved the stochastic robust optimization scheduling problem of a combined cooling, heating, and power integrated energy system using hourly-level supply-demand curves. However, some other studies optimize the system scheduling with a 30-minute optimization scale^13^. However, the IES system generally has strong uncertainty. From the perspective of anti-interference, a smaller scheduling time scale is required. Thus, many single-layer scheduling studies analyze the optimization scheduling problem of the combined cooling, heating, and power integrated energy system with a smaller time scale. Specifically, a typical small-time-scale example is a 15-minute scheduling cycle. For instance, in reference^14^, a scheduling model for a combined cooling, heating, electrical, and gas integrated energy system was established, and 15-minute-level equipment scheduling instructions were obtained by solving this mixed-integer nonlinear problem.

In hierarchical scheduling methods, each layer operates within different scheduling time scales. Generally, the upper layer adopts a longer time scale while the lower layer adopts a smaller one, and the decision-making output of the upper layer serves as the decision-making input for the lower layer. By means of task decomposition and gradually refining the time scale layer by layer, multiple performances of the system, such as economy, reliability, and security, can be taken into account. For example, in reference^15^, 1 h is adopted as the optimization time scale in the upper layer, and reference^16^ also adopts 1 h as the optimization scale. There are also some studies that adopt 15 min, 10 min, etc. as the optimization scheduling scale in the upper-layer scheduling method. After the upper-layer scheduling is completed, in these hierarchical scheduling methods, the upper-layer scheduling results will be used as reference values for the lower-layer scheduling. The lower-layer scheduling method combines short-time-scale prediction values and takes 15 min, 5 min, 2 min, etc. as the optimization cycle for the lower-layer scheduling to correct the upper-layer scheduling results or recalculate for optimized scheduling.

As described above, existing scheduling methods for IES mainly include single-layer and hierarchical scheduling strategies. Each has its own characteristics and limitations. In this context, various studies have been carried out to improve the multi-time-scale coordinated optimization and scheduling considering electric-thermal-cold demand response. The work^17^ developed a multi-time scale demand-controlled operation strategy for the IES. The price-based demand response and indoor temperature-based thermal demand response were both applied to the optimal operation strategy to achieve load shifting and to create flexibility in energy demand. However, the handling of uncertainties in demand and renewable energy generation was relatively simple. Some studies^18,19^ attempted to address these uncertainties by using stochastic programming methods. The authors considered the probabilistic nature of demand and renewable energy, and formulated the optimization problems to minimize the expected cost while satisfying various constraints. However, these methods often suffer from high computational complexity, especially when dealing with a large number of scenarios. This restricts their practical application in real-time operation of IES. To overcome these limitations, data-driven methods have been introduced. For example, deep learning techniques^20,21^ have been used to predict demand and renewable energy generation more accurately. By analyzing historical data, these methods can capture complex patterns and relationships, which helps in better handling uncertainties. Nevertheless, data-driven methods rely heavily on the quality and quantity of historical data. If the data is insufficient or inaccurate, the prediction accuracy will be affected, leading to sub-optimal scheduling results. The works^22^ proposed a multi-timescale power scheduling model considering the coordinated interaction between resources and electrical loads. The work^23^ proposed a multi-timescale scheduling framework for the integrated system of electricity and natural gas at the distribution level. The work^24^ achieved coordinated scheduling between the power system and the regional heating system using an optimal condition decomposition and decentralized solution approach.

To better illustrate how this study fills research gaps and differentiates from prior research, a comparative analysis is presented in Table 1.

**Table 1:** Comparison of the current state of IES research.

Works	Energy resources				Timescales			Demand-side flexibility			
	Electricity	Natural gas	Hydrogen	EV	Day-ahead	Intra-day	Real time	Electricity	Cooling	Heating	EV
Ref.^10^	√	√			√	√		√		√	
Ref.^11^	√	√			√			√	√	√	
Ref.^12^	√	√			√	√	√	√	√	√	
Ref.^13^	√	√	√		√	√		√	√	√	
Ref.^14^	√	√	√		√	√	√	√	√	√	
Ref.^15^	√	√		√	√	√		√	√	√	√
Ref.^16^	√	√			√	√		√	√	√	
Ref.^17^	√	√			√	√		√		√	
Ref.^18^	√	√		√	√	√		√	√	√	√
Ref.^19^	√	√			√	√	√	√	√	√	
Ref.^20^	√	√			√	√		√	√	√	
Ref.^21^	√	√			√	√		√	√	√	
Ref.^22^	√	√		√	√	√	√	√	√	√	√
Ref.^23^	√	√	√	√	√	√	√	√	√	√	√
Ref.^24^	√	√	√	√	√	√	√	√	√	√	√
This paper	√	√	√	√	√	√	√	√	√	√	√

Despite the studies mentioned above considering the impact of temporal scales on system scheduling to varying degrees, they mostly neglect the coordination between the comprehensive energy load response characteristics and system scheduling across different time scales. To address this issue, this paper will focus on how to coordinate scheduling across different time scales considering weather and load forecasting errors, energy response characteristics and adjustment capabilities, and the scheduling costs of flexible loads. This study makes the following contributions:

1. Innovative multi-timescale scheduling: The paper presents a pioneering multi-timescale scheduling approach that integrates and optimizes the operation of generalized energy storage across key operational stages, enhancing the adaptability of integrated energy systems to variability.
2. Comprehensive generalized energy storage integration: It advances the field by formulating a holistic strategy for the inclusion and scheduling of diverse generalized energy storage resources, including emerging technologies, to synergize with demand-side flexibility for operational cost minimization.
3. Demand-side and storage synergy optimization: The research pioneers a novel optimization paradigm that harmonizes demand-side responses with energy storage dynamics, addressing temporal coordination challenges and advancing the efficiency and resilience of integrated energy systems.

The remainder of this paper is organized as follows: Section II introduce the generalized energy storage auxiliary services framework for the IES. Section III presents the proposed multi-timescale optimization scheduling framework, detailing the methodology and algorithms used. Section IV discusses the simulation results, demonstrating the effectiveness of the proposed framework in managing the uncertainties in IES. Finally, Section V concludes the paper and suggests directions for future research.

## Model of integrated energy system

### Framework of generalized energy storage auxiliary service

**Fig. 1:**
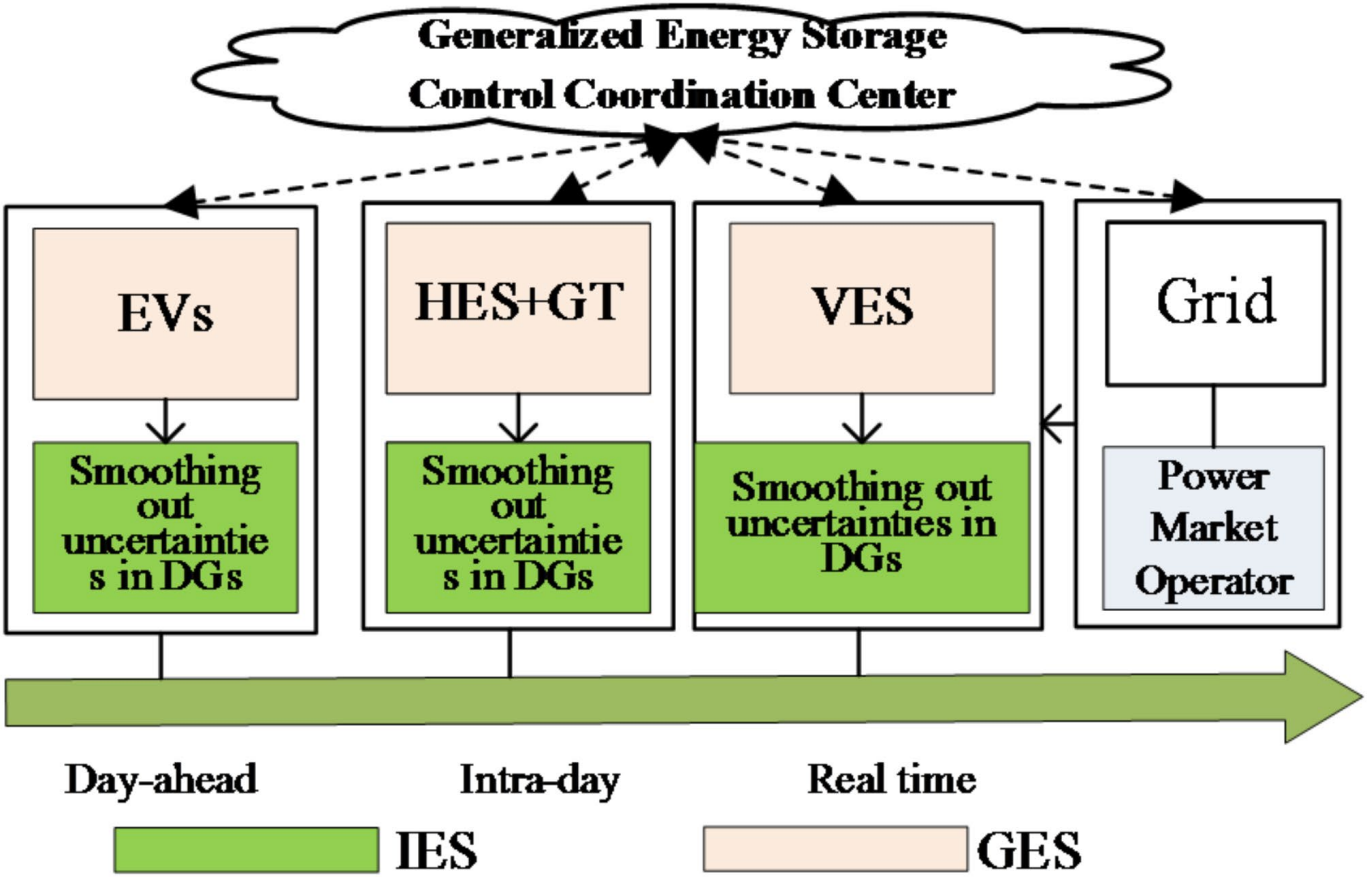
Comprehensive energy system architecture for generalized energy storage auxiliary services.

We describe the bifurcation of generalized energy storage into tangible and virtual energy storage. Virtual energy storage is realized through optimizing controllable load profiles, using virtual parameters to simulate energy storage effects on load balancing. The research aims to utilize generalized energy storage to enhance auxiliary services in integrated energy systems, improving energy efficiency and loosening energy deployment constraints. The study covers different time frames, involving electric vehicles for day-ahead scheduling, hydrogen storage for intraday regulation, and air conditioning clusters’ virtual energy storage for real-time adjustments. The system’s architecture, strengthened by auxiliary services, is shown in Fig. 1.

### Integrated energy system modelling

**Fig. 2:**
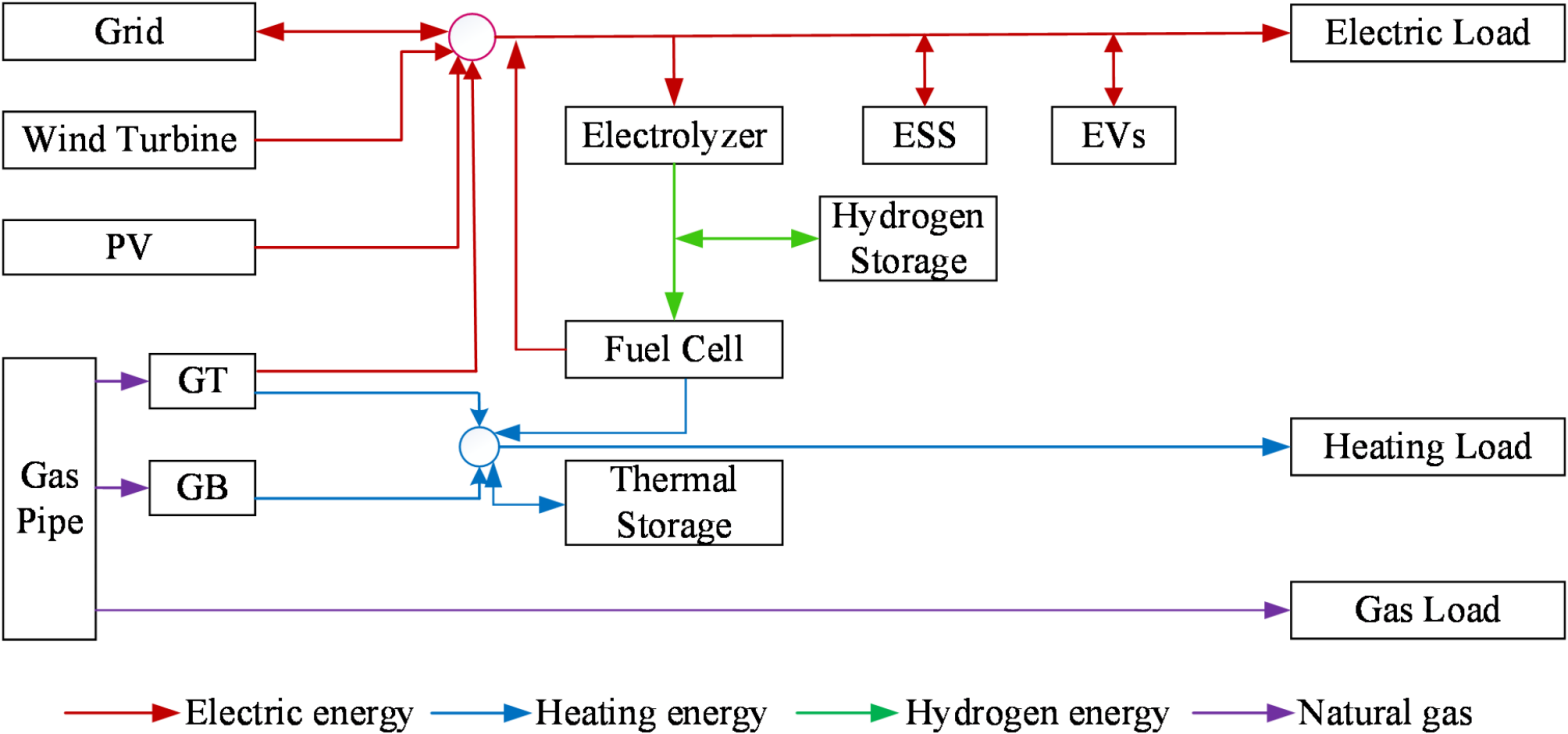
The structure of the integrated energy system .

This study comprehensively considers various energy storage modalities within the integrated energy system. It strategically integrates generalized energy storage resources across different time scales, taking into account their unique attributes, to enhance the system’s ancillary services. The structure of the integrated energy system is given in Fig. 2.

#### Electric energy flow

Wind turbines convert wind energy, PV panels convert solar energy, and gas turbines (GT) generate power from natural gas. All can directly supply the electric load. The grid supplements when needed. Some electricity powers the electrolyzer to produce hydrogen, charges EVs, or is stored in the ESS (electric energy storage) units for later use.

The operation constraints for power generation equipment, ESS equipment, and EVs are as follows:

1 $$P_{{{\text{GT}}}}^{{{\text{min}}}} \leqslant P_{t}^{{{\text{GT}}}} \leqslant P_{{{\text{GT}}}}^{{{\text{max}}}}$$

2 $$- R_{{{\text{GT}}}}^{ - } \leqslant P_{t}^{{{\text{GT}}}} - p_{{t - 1}}^{{{\text{GT}}}} \leqslant R_{{{\text{GT}}}}^{+}$$

3 $$SOC_{{{\text{ev,}}i}}^{{{\text{min}}}} \leqslant SO{C_{{\text{ev}},t,i}} \leqslant SOC_{{{\text{ev,}}i}}^{{{\text{max}}}}$$

4 $$SO{C_{{\text{ev}},i,t}}=SO{C_{{\text{ev}},i,t - 1}}+\eta _{{{\text{ev}}}}^{{{\text{char}}}}P_{{{\text{ev}},i,t}}^{{{\text{char}}}} - P_{{{\text{ev,}}i,t}}^{{{\text{disc}}}}/\eta _{{{\text{ev}}}}^{{{\text{disc}}}}$$

5 $$0 \leqslant P_{{{\text{ev}},i,t}}^{{{\text{char}}}} \leqslant u_{{{\text{ev,}}i,t}}^{{{\text{char}}}}P_{{{\text{ev}},i}}^{{{\text{char,max}}}}$$

6 $$0 \leqslant P_{{{\text{ev}},i,t}}^{{{\text{disc}}}} \leqslant u_{{{\text{ev,}}i,t}}^{{{\text{disc}}}}P_{{{\text{ev}},i}}^{{{\text{disc,max}}}}$$

7 $$u_{{{\text{ev,}}i,t}}^{{{\text{disc}}}}+u_{{{\text{ev,}}i,t}}^{{{\text{char}}}} \leqslant 1$$

where $P_{{{\text{GT}}}}^{{{\text{min}}}}$ and $P_{{{\text{GT}}}}^{{{\text{max}}}}$ are the minimum and maximum output power of the combustion turbine, respectively. $R_{{{\text{GT}}}}^{ - }$ and $R_{{{\text{GT}}}}^{+}$ are the lower and upper limit values of the gas turbine climbing power, respectively. $SO{C_{{\text{ev}},t,i}}$ is the *SOC* state of the *i*-th group of EV in time period *t* after clustering. $SOC_{{{\text{ev,}}i}}^{{{\text{min}}}}$ and $SOC_{{{\text{ev,}}i}}^{{{\text{max}}}}$ are the lower and upper limit values of the battery charging state of the electric vehicle in group *i*, respectively. $p_{{{\text{ev}},i,t}}^{{{\text{char}}}}$ and $p_{{{\text{ev}},i,t}}^{{{\text{disc}}}}$ are the charging and discharging power of *i-*th group of EVs in the time period, respectively. $\eta _{{{\text{ev}}}}^{{{\text{char}}}}$ and $\eta _{{{\text{ev}}}}^{{{\text{disc}}}}$ are the are the charging and discharging efficiency coefficients of the electric vehicle battery, respectively. $P_{{{\text{ev}},i}}^{{{\text{char,max}}}}$ and $P_{{{\text{ev}},i}}^{{{\text{disc,max}}}}$ are the maximum charging and discharging power of the i-th electric vehicle battery, respectively. $u_{{{\text{ev,}}i,t}}^{{{\text{disc}}}}$ and $u_{{{\text{ev,}}i,t}}^{{{\text{char}}}}$ are the 0–1 variables, correspond to the charging and discharging state of the *i*-th group of EVs at time period *t*. Charging is taken as 1,otherwise it is taken as 0.

The electric power balance in IES is as follows:

8 $$P_{t}^{{{\text{gt}}}}+P_{t}^{{{\text{PV}}}}+P_{t}^{{{\text{WT}}}}+P_{t}^{{{\text{edis}}}}+\sum\limits_{{i=1}}^{N} {P_{{{\text{ev,}}i{\text{,}}t}}^{{{\text{disc}}}}} +P_{t}^{{{\text{buy}}}}+P_{t}^{{{\text{H2e}}}}=P_{t}^{{{\text{sell}}}}+\sum\limits_{{i=1}}^{N} {P_{{{\text{ev,}}i,t}}^{{{\text{char}}}}} +P_{t}^{{{\text{echa}}}}+P_{t}^{{{\text{el}}}}+P_{t}^{{{\text{e2H}}}}$$

where $P_{t}^{{{\text{WT}}}}$ and $P_{t}^{{{\text{PV}}}}$ are are the wind power and photovoltaic output power. $P_{t}^{{{\text{echa}}}}$ and $P_{t}^{{{\text{edis}}}}$ are the charging and discharging power of electric energy storage units. $P_{t}^{{{\text{el}}}}$ is the electric load. $P_{t}^{{{\text{e2H}}}}$ is the power of hydrogen production from electrolyzed water. $P_{t}^{{{\text{H2e}}}}$ is the power generated by the hydrogen fuel cell.

#### Heating energy flow

The heating load is served by thermal energy. The GT and gas-fired boiler (GB) in the system can convert natural gas into thermal energy. This thermal energy can be directly used to meet the heating load or stored in the thermal storage for later use.

The operation constraints regarding heat-generating equipment and heat-storage equipment are as follows:

9 $$Q_{{{\text{GB}}}}^{{{\text{min}}}} \leqslant Q_{t}^{{{\text{GB}}}} \leqslant Q_{{{\text{GB}}}}^{{{\text{max}}}}$$

10 $$- R_{{{\text{GB}}}}^{ - } \leqslant p_{t}^{{{\text{gb}}}} - p_{{t - 1}}^{{{\text{gb}}}} \leqslant R_{{{\text{GB}}}}^{+}$$

11 $$Q_{t}^{{{\text{GT}}}}+Q_{t}^{{{\text{GB}}}}+Q_{t}^{{{\text{hdis}}}}=Q_{t}^{{{\text{hcha}}}}+Q_{t}^{{{\text{hl}}}}+Q_{t}^{{{\text{qh}}}}$$

where $Q_{{{\text{GB}}}}^{{{\text{min}}}}$ and $Q_{{{\text{GB}}}}^{{{\text{max}}}}$ are the minimum and maximum output power of the gas-fired boiler, respectively. $R_{{{\text{GB}}}}^{ - }$ and $R_{{{\text{GB}}}}^{+}$ are the lower and upper limit values of the gas-fired boiler climbing power, respectively. $Q_{t}^{{{\text{hcha}}}}$ and $Q_{t}^{{{\text{hdis}}}}$ are the storing power and releasing power of the heat storage device, respectively. $Q_{t}^{{{\text{qh}}}}$ is the heating load power.

#### Hydrogen energy flow

Hydrogen is produced by the electrolyzer, which uses electricity to split water into hydrogen and oxygen. The produced hydrogen is then stored in the hydrogen storage. When needed, the hydrogen can be fed into the fuel cell to generate electricity, which can be used to meet the electric load.

12 $$m_{t}^{{{{\text{H}}_{\text{2}}}}}=m_{{t - 1}}^{{{{\text{H}}_{\text{2}}}}}+\frac{{{\eta ^{{\text{ELZ}}}}P_{{t - 1}}^{{{\text{e2H}}}}}}{{{k_{{\text{HHV}}}}}} - \frac{{P_{{t - 1}}^{{{\text{H2e}}}}}}{{{\eta ^{{\text{FC}}}}{k_{{\text{HHV}}}}}} - m_{{t - 1}}^{{{{\text{H}}_{\text{2}}}{\text{,ld}}}}$$

13 $$\left\{ {\begin{array}{*{20}{c}} {0 \leqslant P_{t}^{{{\text{e2H}}}} \leqslant \mu _{t}^{{{\text{e2H}}}}P_{{\hbox{max} }}^{{{\text{e2H}}}}} \\ {0 \leqslant P_{t}^{{{\text{H2e}}}} \leqslant \mu _{t}^{{{\text{H2e}}}}P_{{\hbox{max} }}^{{{\text{H2e}}}}} \\ {\mu _{t}^{{{\text{e2H}}}}+\mu _{t}^{{{\text{H2e}}}} \leqslant 1} \end{array}} \right.$$

where $m_{t}^{{{{\text{H}}_{\text{2}}}}}$ and $m_{{t - 1}}^{{{{\text{H}}_{\text{2}}}}}$ denote the mass of hydrogen in the hydrogen storage tank at time periods *t* and *t*-1, kg. $m_{{t - 1}}^{{{{\text{H}}_{\text{2}}}{\text{,ld}}}}$ denotes the mass of the hydrogen load at time period *t*-1, kg. $P_{t}^{{{\text{e2H}}}}$ is the power of hydrogen production from electrolyzed water at time period t, kW. $P_{t}^{{{\text{H2e}}}}$ is the power generated by the hydrogen fuel cell at time period t, kW. ${\eta ^{{\text{ELZ}}}}$ and ${\eta ^{{\text{FC}}}}$ are denote the efficiency of water electrolysis and fuel cell power generation, respectively. ${k_{{\text{HHV}}}}$ denotes the calorific value of hydrogen, 3.75 kWh/m^3^. μ_t_^e2H^ and μ_t_^H2e^are 0/1 variables, to avoid simultaneous hydrogen production and power generation.

#### Gas flow

Natural gas is supplied from the gas pipe. It can be used as a fuel for the GT and GB to generate thermal energy for the heating load. Also, natural gas can be directly supplied to the gas load to meet its demand. The supply and demand balance constraint for natural gas is as follows:

14 $$G_{t}^{{{\text{ch4}}}}=G_{t}^{{{\text{cgt}}}}+G_{t}^{{{\text{cgb}}}}+G_{t}^{{{\text{ql}}}}$$

where $G_{t}^{{{\text{cgt}}}}$ and $G_{t}^{{{\text{cgb}}}}$ are the gas consumption of gas turbines and gas boilers, respectively. $G_{t}^{{{\text{ch4}}}}$ and $G_{t}^{{{\text{ql}}}}$ are the total natural gas consumption and the natural gas load, respectively.

#### EV stochastic behavior

In this work, arrival time, departure time and travel distance are three attributes related to driving behavior. The power exchange profile of EVs depends on the arrival time, departure time and distance travelled by the vehicles.

In order to capture the uncertainty in driving behavior, the uncertainty in EV arrival time *t*_arr_, departure time *t*_dep_, and daily travel distance *d*, using Monte Carlo Simulation (MCS)^25^. MCS is a computational algorithm that relies on repeated random sampling to obtain numerical results. Here, it is used to generate a large number of possible combinations of arrival time, departure time, and travel distance to account for their uncertainties.

- Arrival/Departure Times: These are modeled as normal distributions ***N*** (*µ*, *σ*^2^), where *t*_arr_=17:00, *µ*_arr_=1.5 h; *t*_dep_=8:00, *µ*_dep_=1.2 h.
- Travel Distance: Modeled using a log-normal distribution Log-***N*** (*µ* = 3.2, *σ =* 0.8) derived from the NHTS 2009 database^26^.

Then, 500 scenarios of EV driving patterns are generated by using MCS. After that, the simultaneous backward reduction technique is applied. This technique helps in reducing the complexity of the analysis while still retaining the key characteristics of the original set of scenarios.

## Multi-timescale optimal scheduling model for IES

### Day-ahead optimal scheduling model

#### Objective function of day-head stage

The objective function consists of five components, namely, the cost of purchasing natural gas ${\text{C}}_{t}^{{{\text{ng}}}}$, the cost of compensating electric vehicle users ${\text{C}}_{t}^{{{\text{ev}}}}$, the environmental cost of gas turbines ${\text{C}}_{t}^{{{\text{env}}}}$, the revenue from the sale of electricity by the integrated energy entity to the higher-level grid ${\text{C}}_{t}^{{{\text{el}}}}$, and the cost of operation and maintenance of hydrogen storage ${\text{C}}_{t}^{{{\text{OMH2}}}}$.

15 $$\hbox{min} \sum\limits_{{t=1}}^{{24}} {({\text{C}}_{t}^{{{\text{ng}}}}+{\text{C}}_{t}^{{{\text{ev}}}}+{\text{C}}_{t}^{{{\text{env}}}}+{\text{C}}_{t}^{{{\text{el}}}}+{\text{C}}_{t}^{{{\text{OMH2}}}})}$$

The specific expressions for each cost component of the objective function are as follows.

16 $${\text{C}}_{t}^{{{\text{ng}}}}=R_{t}^{{{\text{ng}}}}(P_{t}^{{{\text{gl}}}}+P_{t}^{{{\text{gt}}}}/{\eta _{{\text{gt}}}}+P_{t}^{{{\text{gb}}}}/{\eta _{{\text{gb}}}})$$

17 $$C_{t}^{{ev}} = e_{{ev}} \sum\limits_{{i = 1}}^{N} {P_{{ev,i,t}}^{{disc}} } ,t \in T^{{Grid}}$$

18 $$C_{t}^{{env}} = P_{t}^{{gt}} \sum\limits_{{l = 1}}^{n} {Q_{l} } (Y_{l} + B_{l} )$$

19 $${\text{C}}_{t}^{{{\text{el}}}}=P_{t}^{{{\text{buy}}}}c_{t}^{{{\text{eg}}}} - P_{t}^{{{\text{sell}}}}c_{t}^{{\text{e}}}$$

20 $${\text{C}}_{t}^{{{\text{OMH2}}}}={\eta ^{{\text{OMH2}}}}(p_{t}^{{{\text{e2H}}}}+p_{t}^{{{\text{H2e}}}})$$

In expression (16), $R_{t}^{{{\text{ng}}}}$ denotes the price of natural gas, CNY/MWh. $P_{t}^{{{\text{gl}}}}$ the natural gas load. $P_{t}^{{{\text{gt}}}}$ denotes gas turbine power output. ${\eta _{{\text{gt}}}}$ is the gas turbine gas-electric efficiency factor. $P_{t}^{{{\text{gb}}}}$ denotes the thermal power output of the boiler. ${\eta _{{\text{gb}}}}$ is the coefficient of thermal efficiency of the boiler. In expression (17), ${e_{{\text{ev}}}}$ denotes coefficients for the compensation of electric vehicle users by integrated energy system. ${P_{{\text{disc}},t}}$ denotes the EV discharge power. *N* denotes the number of electric vehicle clusters formed by NJW spectral clustering. ${T^{{\text{Grid}}}}$ is time period for grid integration of electric vehicles. In expression (18), ${Q_l}$ denotes the emission intensity of the gas turbine’s *l*-th pollutant gas. ${Y_l}$ and ${B_l}$ are penalty cost coefficients and environmental value cost coefficients, respectively, of the *l*-th pollutant gas. In expression (19), $P_{t}^{{{\text{sell}}}}$ and $P_{t}^{{{\text{buy}}}}$ are the sale and purchase of electric power to the higher grid for the integrated energy system. $c_{t}^{{\text{e}}}$ and $c_{t}^{{{\text{eg}}}}$ are the tariff for the sale and purchase of electricity from the integrated energy system to the higher grid. In expression (20), $P_{t}^{{{\text{e2H}}}}$ and $P_{t}^{{{\text{H2e}}}}$ are the electric hydrogen production from hydrogen storage systems and electric power generation from hydrogen fuel cells, respectively. ${\eta ^{{\text{OMH2}}}}$ denotes the cost factor for operation and maintenance of hydrogen energy storage systems.

#### Constraints of day-head stage

The day-ahead robust optimization scheduling model of IES contains electric energy flow constraints, heating energy flow constraints, hydrogen energy flow constraints, and natural gas flow constraints, as shown in (1)–(14).

#### Optimization models considering uncertainty

A pivotal consideration within this optimization model is the stochastic nature of renewable energy generation, which introduces a variable that can incur substantial penalty costs due to deviations between forecast output and actual output^27^. The study posits renewable energy generation as an autonomous participant within the IES, endowed with the agency to determine its own output levels. The core objective of this model is the minimization of the IES’s operational expenditures, thereby establishing a cost-minimization function that encapsulates the strategic interplay between the renewable energy participant and the overarching IES.

21 $$\mathop {{\text{min}}{\kern 1pt} {\kern 1pt} }\limits_{{{\Psi _1}}} \mathop {{\text{max}}}\limits_{{\tilde {P}_{t}^{{{\text{PV}}}}{\text{,}}\tilde {P}_{t}^{{{\text{WT}}}}}} {\kern 1pt} \sum\limits_{{t=1}}^{T} {(C_{t}^{{{\text{ng}}}}+{\text{C}}_{t}^{{{\text{ev}}}}+{\text{C}}_{t}^{{{\text{env}}}}+{\text{C}}_{t}^{{{\text{el}}}}+{\text{C}}_{t}^{{{\text{OMH2}}}}+c_{t}^{+}\Delta P_{t}^{+}+c_{t}^{ - }\Delta P_{t}^{ - })}$$

s.t. Expression (7)–(21)

22 $$\Delta P_{t}^{+}={\text{max}}{\kern 1pt} {\kern 1pt} {\kern 1pt} {\kern 1pt} {\kern 1pt} {\text{(}}P_{t}^{{{\text{PV}}}}+P_{t}^{{{\text{WT}}}} - \tilde {P}_{t}^{{{\text{PV}}}} - \tilde {P}_{t}^{{{\text{WT}}}},0)$$

23 $$\Delta P_{t}^{ - }={\text{max}}{\kern 1pt} {\kern 1pt} {\kern 1pt} {\kern 1pt} {\kern 1pt} {\text{(}}\tilde {P}_{t}^{{{\text{PV}}}}+\tilde {P}_{t}^{{{\text{WT}}}} - P_{t}^{{{\text{PV}}}} - P_{t}^{{{\text{WT}}}},0)$$

24 $$\tilde {P}_{t}^{{{\text{PV}}}}=P_{t}^{{{\text{PV}}}}+z_{t}^{{{\text{PV}}}}{\kern 1pt} {\kern 1pt} {\kern 1pt} {\kern 1pt} {\kern 1pt} {\kern 1pt} {\kern 1pt} ,{\kern 1pt} {\kern 1pt} {\kern 1pt} {\kern 1pt} {\kern 1pt} {\kern 1pt} {\kern 1pt} z_{t}^{{{\text{PV}}}} \in {\kern 1pt} {\kern 1pt} [ - 0.15P_{t}^{{{\text{PV}}}},0.15P_{t}^{{{\text{PV}}}}]$$

25 $$\sum\limits_{{t=1}}^{{24}} {\left| {\frac{{\tilde {P}_{t}^{{{\text{PV}}}} - P_{t}^{{{\text{PV}}}}}}{{P_{t}^{{{\text{PV}}}}}}} \right|} \leqslant {\Gamma _{{\text{PV}}}}$$

26 $$\tilde {P}_{t}^{{{\text{WT}}}}=P_{t}^{{{\text{WT}}}}+z_{t}^{{{\text{WT}}}}{\kern 1pt} {\kern 1pt} {\kern 1pt} ,{\kern 1pt} {\kern 1pt} {\kern 1pt} {\kern 1pt} {\kern 1pt} z_{t}^{{{\text{WT}}}} \in {\kern 1pt} {\kern 1pt} [ - 0.15P_{t}^{{{\text{WT}}}},0.15P_{t}^{{{\text{WT}}}}]$$

27 $$\sum\limits_{{t=1}}^{{24}} {\left| {\frac{{\tilde {P}_{t}^{{{\text{WT}}}} - P_{t}^{{{\text{WT}}}}}}{{P_{t}^{{{\text{WT}}}}}}} \right|} \leqslant {\Gamma _{{\text{WT}}}}$$

where ${\Psi _1}$ is the set of decision variables that synthesize the output of each device within the IES. $\tilde {P}_{t}^{{{\text{PV}}}}$ and $\tilde {P}_{t}^{{{\text{WT}}}}$ are the actual output of distributed renewable energy generation. The term $c_{t}^{+}\Delta P_{t}^{+}$ denotes the penalty cost of purchasing additional electricity due to overestimation of renewable energy output. $c_{t}^{+}$ is the penalty price for purchasing electricity after overestimation, which is 1.5 times the purchase price of electricity. $\Delta P_{t}^{+}$ denotes the is the overestimated output power of renewable energy generating units. On the contrary, the term $c_{t}^{ - }\Delta P_{t}^{ - }$ denotes the penalty cost of purchasing additional electricity due to underestimation of renewable energy output. $c_{t}^{ - }$ is the penalty price for purchasing electricity after underestimation, which is 1.5 times the sold price of electricity. $\Delta P_{t}^{ - }$ denotes the is the underestimation output power of renewable energy generating units. $P_{t}^{{{\text{PV}}}}$ and $P_{t}^{{{\text{WT}}}}$ denote the predicted value of wind power and photovoltaic power in the day-ahead stage. $z_{t}^{{{\text{PV}}}}{\kern 1pt}$ and ${\kern 1pt} {\kern 1pt} z_{t}^{{{\text{WT}}}}$ denote the deviation of wind power and photovoltaic power. ${\Gamma _{{\text{PV}}}}$ and ${\Gamma _{{\text{WT}}}}$ are both robustness factor.

To address the min-max optimization problem as delineated in Expression (20), this study employs the Column-and-Constraint Generation (C&CG) method to decompose the problem into a master problem and a series of subproblems, which are solved iteratively.

### Intra-day optimal scheduling model

The integrated energy system optimizes its day-ahead scheduling to submit power declarations to the grid operator, transitioning equipment status updates to the intraday stage. Utilizing more precise predictions from the prior hour’s operations, the intraday stage allows for the re-optimization of gas turbines and hydrogen storage systems, incorporating refined wind and solar data alongside alternative and transferable loads, to stabilize renewable energy output fluctuations.

#### Flexible load

Within an integrated energy system, a hierarchy of energy resources is interconverted through transformational equipment, facilitating the reciprocal substitution and utilization of diverse energy forms. Anchored in the concept of multi-energy coupling, the substitutional loads comply with the law of conservation of energy, delineating their conversion relationships as follows:

28 $$\Delta {R_i}= - {\gamma _{ij}}\Delta {R_j}$$

29 $${\gamma _{ij}}=\frac{{{M_i}{k_i}}}{{{M_j}{k_j}}}$$

30 $$0 \leqslant \Delta {R_i} \leqslant \Delta R_{i}^{{\hbox{max} }}$$

31 $$0 \leqslant \Delta {R_j} \leqslant \Delta R_{j}^{{\hbox{max} }}$$

where *i* and *j* are for any two of the three energy sources: electricity, heat, and natural gas, respectively. $\Delta {R_i}$ and $\Delta {R_j}$ are the amount of change in any two energy sources, respectively. *M*_*i*_ and *M*_*j*_ correspond to the calorific value of the two energy sources and the energy source, respectively. ${k_i}$ and ${k_j}$ correspond to the utilization of the two energy sources, respectively. $\Delta R_{i}^{{\hbox{max} }}$ and $\Delta R_{j}^{{\hbox{max} }}$ correspond to the maximum change in these two energy sources, respectively. The electric heat and natural gas load after energy substitution is ${P_i}=P_{i}^{0}+\Delta {R_i}$.

Drawing upon the electricity pricing data released in the intraday market, the user’s energy consumption is meticulously recalibrated in accordance with the fluctuating real-time electricity rates. Succinctly, this strategy involves the strategic postponement of non-essential electrical loads to periods characterized by reduced electricity pricing or cost parity, thereby achieving a more cost-effective energy usage pattern. The load transfer model is encapsulated by the following principles:

32 $$\sum\limits_{{t=1}}^{T} {P_{t}^{{{\text{sl}}}}} =d\sum\limits_{{t=1}}^{T} {P_{t}^{{{\text{el}}}}}$$

33 $$0 \leqslant P_{t}^{{{\text{sl}}}} \leqslant k \times P_{t}^{{{\text{el}}}}$$

where $P_{t}^{{{\text{sl}}}}$ is the size of transferable load power in time period *t*. In this study, the total amount of transferable load is equal to the total amount of load in a certain proportion *d*, and the transferable load power in each moment is not greater than *k* times of the load before regulation.

#### Objective function of intraday stage

During the intraday stage, the IES employs the auxiliary support of gas turbines and hydrogen energy storage systems to refine its optimization process with more precise data on renewable energy generation. This strategy also takes into account the participation of demand response loads. The aim is to minimize the deviation in the declared optimization outcomes from actual performance while enhancing the system’s energy utilization flexibility and economic viability. The objective function for this stage is delineated as:

34 $$\hbox{min} \sum\limits_{{t=1}}^{{24}} {(\Delta {\text{C}}_{t}^{{{\text{ng}}}}+\Delta {\text{C}}_{t}^{{{\text{env}}}}+{\text{C}}_{t}^{{{\text{pun}}}}+{\text{C}}_{t}^{{{\text{res}}}}+{\text{C}}_{t}^{{{\text{sl}}}}+\Delta {\text{C}}_{t}^{{{\text{OMH2}}}})}$$

where $\Delta {\text{C}}_{t}^{{{\text{ng}}}}$ and $\Delta {\text{C}}_{t}^{{{\text{env}}}}$ denote the incremental cost of gas purchase and environmental protection in the mid-day stage, respectively. ${\text{C}}_{t}^{{{\text{pun}}}}$ and ${\text{C}}_{t}^{{{\text{res}}}}$ denote the new energy output declaration deviation penalty cost and the alternative load penalty cost. ${\text{C}}_{t}^{{{\text{sl}}}}$ denotes the transferable load penalty cost. $\Delta {\text{C}}_{t}^{{{\text{OMH2}}}}$ denotes the incremental hydrogen storage O&M cost.

The cost of each part of the objective function in the intraday optimization stage are expressed as follows:

35 $$\Delta {\text{C}}_{t}^{{{\text{ng}}}}=R_{t}^{{{\text{ng}}}}(\Delta P_{t}^{{{\text{gl}}}}+\Delta P_{t}^{{{\text{gt}}}}/{\eta _{{\text{gt}}}}+\Delta P_{t}^{{{\text{gb}}}}/{\eta _{{\text{gb}}}})$$

36 $$\Delta C_{t}^{{env}} = \Delta P_{t}^{{gt}} \sum\limits_{{l = 1}}^{n} {Q_{l} } (Y_{l} + B_{l} )$$

37 $${\text{C}}_{t}^{{{\text{pun}}}}=c_{t}^{{z+}}\Delta P_{t}^{{z+}}+c_{t}^{{z - }}\Delta P_{t}^{{{\text{z}} - }}$$

38 $$\Delta P_{t}^{{z + }} = \max (P_{t}^{{qgr}} - P_{t}^{{zgr}} ,0)$$

39 $$\Delta P_{t}^{{z - }} = \max (P_{t}^{{zgr}} - P_{t}^{{qgr}} ,0)$$

40 $${\text{C}}_{t}^{{{\text{res}}}}={R^{{\text{el}}}}\left| {\Delta P_{t}^{{{\text{el}}}}} \right|+{R^{{\text{hl}}}}\left| {\Delta P_{t}^{{{\text{hl}}}}} \right|+{R^{{\text{ql}}}}\left| {\Delta P_{t}^{{{\text{ql}}}}} \right|$$

41 $${\text{C}}_{t}^{{{\text{sl}}}}={{\text{c}}^{{\text{sl}}}}P_{t}^{{{\text{sl}}}}$$

42 $$\Delta {\text{C}}_{t}^{{{\text{OMH2}}}}={\eta ^{{\text{OMH2}}}}(\Delta P_{t}^{{{\text{e2H}}}}+\Delta P_{t}^{{{\text{H2e}}}})$$

where $\Delta P_{t}^{{{\text{gl}}}}$, $\Delta P_{t}^{{{\text{gt}}}}$ and $\Delta p_{t}^{{{\text{gb}}}}$ denote represent the natural gas load, g as turbine output, and boiler heat production power increments, respectively, during the intraday optimization stage of operation. $P_{t}^{{{\text{qgr}}}}$ and $P_{t}^{{{\text{zgr}}}}$ represent the power interactions with the higher grid during the day-ahead and intraday stages, respectively. ${R^{{\text{el}}}}$, ${R^{{\text{hl}}}}$ and ${R^{{\text{ql}}}}$ represent the unit compensation cost coefficients for involved substitution for electric, thermal, and natural gas loads, respectively. $\Delta p_{t}^{{{\text{el}}}}$, $\Delta p_{t}^{{{\text{hl}}}}$ and $\Delta p_{t}^{{{\text{ql}}}}$ represent represent the load of electricity, heat, and natural gas, respectively, involved in the substitution. ${{\text{c}}^{{\text{sl}}}}$ represents unit compensation cost factor for transferable load.

#### Constraints of intraday stage

In the Intra-day optimization model for hydrogen storage participation in auxiliary services optimization model, the constraints that need to be satisfied include the previously mentioned constraints (7)–(10), as well as the alternative load response constraints, the integrated energy in the intra-day stage Multi-energy supply and demand balance constraints within the system, as shown in expressions (43)–(48).

43 $$- R_{{\text{e}}}^{ - }P_{t}^{{{\text{el}}}} \leqslant \Delta P_{t}^{{{\text{el}}}} \leqslant R_{{\text{e}}}^{+}P_{t}^{{{\text{el}}}}$$

44 $$- R_{{\text{h}}}^{ - }P_{t}^{{{\text{hl}}}} \leqslant \Delta P_{t}^{{{\text{hl}}}} \leqslant R_{{\text{h}}}^{+}P_{t}^{{{\text{hl}}}}$$

45 $$- R_{{\text{q}}}^{ - }P_{t}^{{{\text{ql}}}} \leqslant \Delta P_{t}^{{{\text{ql}}}} \leqslant R_{{\text{q}}}^{+}P_{t}^{{{\text{ql}}}}$$

46 $$\begin{gathered} P_{t}^{{{\text{gt}}}}+P_{t}^{{{\text{PV,z}}}}+P_{t}^{{{\text{WIN,z}}}}+P_{t}^{{{\text{edis}}}}+\sum\limits_{{i=1}}^{N} {P_{{{\text{ev,}}i{\text{,}}t}}^{{{\text{disc}}}}} +P_{t}^{{{\text{buy}}}}+P_{t}^{{{\text{H2e}}}} \hfill \\ \begin{array}{*{20}{c}} {}&{}&{}&{} \end{array}=P_{t}^{{{\text{sell}}}}+\sum\limits_{{i=1}}^{N} {P_{{{\text{ev,}}i,t}}^{{{\text{char}}}}} +P_{t}^{{{\text{echa}}}}+0.8P_{t}^{{{\text{el}}}}+\Delta P_{t}^{{{\text{el}}}}+P_{t}^{{{\text{H2e}}}}+P_{t}^{{{\text{sl}}}} \hfill \\ \end{gathered}$$

47 $$Q_{t}^{{{\text{gh}}}}+Q_{t}^{{{\text{gb}}}}+\Delta Q_{t}^{{{\text{gb}}}}+Q_{t}^{{{\text{hdis}}}}=Q_{t}^{{{\text{hcha}}}}+Q_{t}^{{{\text{hl}}}}+Q_{t}^{{{\text{qh}}}}+\Delta Q_{t}^{{{\text{qh}}}}$$

48 $$G_{t}^{{{\text{zch4}}}}=G_{t}^{{{\text{cgt}}}}+G_{t}^{{{\text{cgb}}}}+G_{t}^{{{\text{ql}}}}+\Delta G_{t}^{{{\text{ql}}}}$$

where $R_{{\text{e}}}^{ - }$ and $R_{{\text{e}}}^{+}$ represent the maximum scale factor for conversion of electrical energy to other forms of energy and other forms of energy to electrical energy, respectively. $R_{{\text{h}}}^{ - }$ and $R_{{\text{h}}}^{+}$ represent the maximum scale factor for conversion of heating energy to other forms of energy and other forms of energy to heating energy, respectively. $R_{{\text{q}}}^{ - }$ and $R_{{\text{q}}}^{+}$ represent the maximum scale factor for conversion of hydrogen energy to other forms of energy and other forms of energy to hydrogen energy, respectively. $P_{t}^{{{\text{PV,z}}}}$ and $p_{t}^{{{\text{WT,z}}}}$ denote the predicted values of PV and wind power generation in the intraday optimization stage, respectively. $G_{t}^{{{\text{zch4}}}}$ denotes the total gas consumption of the light energy complex during the intraday optimization stage.

### Real-time optimal scheduling model

#### Basics of virtual energy storage for air conditioning

The extant research on air conditioning load modeling is extensive and can be broadly divided into two principal methodologies. One methodology is founded on the law of conservation of energy to formulate air conditioning load models. The other methodology employs equivalent thermal parameters (ETP) models^20^. This paper adopts a first-order ETP equivalent thermal model for the analysis.

49 $$\frac{{{\text{d}}{T_{{\text{room,}}t}}}}{{{\text{d}}t}}= - \frac{{{Q_{{\text{ac,}}t}}}}{{{C_{{\text{eq}}}}}}+\frac{{{T_{{\text{out,}}t}} - {T_{{\text{room,}}t}}}}{{{R_{{\text{eq}}}}{C_{{\text{eq}}}}}}$$

where ${T_{{\text{room,}}t}}$ represents the indoor temperature in time period *t*;${T_{{\text{out,}}t}}$ denotes the outdoor temperature in time period *t*; ${Q_{{\text{ac,}}t}}$ signifies the cooling capacity of the air conditioning system in time period *t*; ${C_{{\text{eq}}}}$ and ${R_{{\text{eq}}}}$ denote the equivalent heat capacity and equivalent thermal resistance of the building, respectively.

The cooling power of an air conditioning unit is related to the power consumption as follows:

50 $${Q_{{\text{ac,}}t}}=\lambda {P_{{\text{ac,}}t}}$$

where $\lambda$ the cooling efficiency of air conditioner.

In a sufficiently short period of time, it can be assumed that the outdoor temperature remains constant. Assuming that the indoor temperature is at the most comfortable level at this time, the air conditioner is in a steady state, and the following relationship can be obtained:

51 $$\frac{{{\text{d}}{T_{{\text{room,}}t}}}}{{{\text{d}}t}}=0$$

According to equations (49) to (51), the following relationships hold:

52 $${P_{{\text{ac0,}}t}}=\frac{{{T_{{\text{out,}}t}} - {T_{{\text{comf,}}t}}}}{{\lambda {R_{{\text{eq}}}}}}$$

**Fig. 3:**
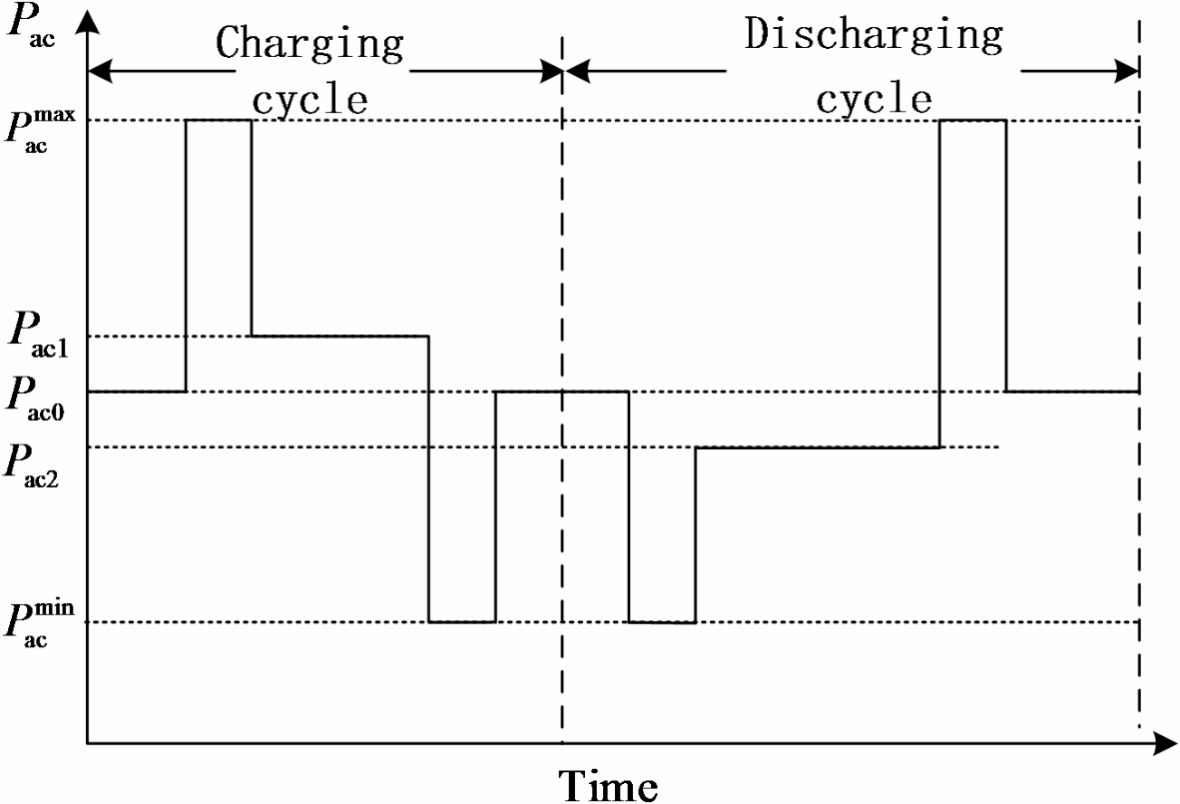
Air conditioning load power change curve.

At a certain moment, the indoor set temperature is ${T_{{\text{set}}}}$, which is higher or lower than the most comfortable temperature ${T_{{\text{comf}}}}$, causing the air conditioner to operate in a transient state. As shown in Fig. 3, if ${T_{{\text{set1}}}}$<${T_{{\text{comf}}}}$, the air conditioner runs at the maximum power $P_{{{\text{ac}}}}^{{{\text{max}}}}$. When the indoor temperature reaches ${T_{{\text{set1}}}}$, the air conditioner enters a transient mode, operating at power $P_{{{\text{ac1}}}}^{{}}$ to maintain the indoor temperature at ${T_{{\text{set1}}}}$; if ${T_{{\text{set2}}}}$>${T_{{\text{comf}}}}$, the air conditioner runs at the minimum power $P_{{{\text{ac}}}}^{{{\text{min}}}}$. When the indoor temperature reaches ${T_{{\text{set1}}}}$, the air conditioner enters a transient mode, operating at a certain power to maintain the indoor temperature. Since the response rate of residential air conditioners is less than 5 min, which is shorter than the integrated energy dispatch cycle, to ensure continuous operation in each dispatch cycle, the indoor temperature needs to be adjusted back to the most comfortable temperature ${T_{{\text{comf}}}}$ at the end of the dispatch cycle. Similarly, if ${T_{{\text{set2}}}}$<${T_{{\text{comf}}}}$, the air conditioner runs at the maximum power $P_{{{\text{ac}}}}^{{{\text{max}}}}$. When the indoor temperature reaches ${T_{{\text{set2}}}}$, the air conditioner enters a transient mode, operating at power $P_{{{\text{ac2}}}}^{{}}$ to maintain the indoor temperature at ${T_{{\text{set2}}}}$. Similarly, at the end of the dispatch cycle, the room temperature needs to be readjusted back to ${T_{{\text{comf}}}}$ to ensure the sustainability of the dispatch cycle.

53 $${P_{{\text{ac1,}}t}}=\frac{{{T_{{\text{out,}}t}} - {T_{{\text{set1,}}t}}}}{{\lambda {R_{{\text{eq}}}}}}$$

54 $${P_{{\text{ac2,}}t}}=\frac{{{T_{{\text{out,}}t}} - {T_{{\text{set2,}}t}}}}{{\lambda {R_{{\text{eq}}}}}}$$

Assuming a dispatch cycle is denoted as $\Delta t$, and it is known that the air conditioner operates at $P_{{{\text{ac}}}}^{{{\text{max}}}}$ for a time duration $\Delta {t_1}$, and at $P_{{{\text{ac}}}}^{{{\text{min}}}}$ for a time duration $\Delta {t_2}$. When the air conditioner is in the charging cycle, the calculation formulas for the charging times are as shown in Eqs. (55) and (56), and the maximum charging power of the air conditioner’s virtual energy storage is $P_{{{\text{con}}}}^{{{\text{cha}}}}$ as indicated in Eq. (57); when the air conditioner is in the discharging cycle, the calculation formulas for the discharging times are as shown in Eqs. (58) and (59), and the maximum discharging power of the air conditioner’s virtual energy storage is $P_{{{\text{con}}}}^{{{\text{dis}}}}$ as indicated in expression (60).

55 $$\Delta t_{1}^{{{\text{cha}}}}={R_{{\text{eq}}}}{C_{{\text{eq}}}}{\text{ln}}\frac{{{T_{{\text{comf}}}} - {T_{{\text{out}}}}+\lambda {P_{\hbox{max} }}{R_{{\text{eq}}}}}}{{{T_{{\text{set1}}}} - {T_{{\text{out}}}}+\lambda {P_{\hbox{max} }}{R_{{\text{eq}}}}}}$$

56 $$\Delta t_{2}^{{{\text{cha}}}}={R_{{\text{eq}}}}{C_{{\text{eq}}}}{\text{ln}}\frac{{{T_{{\text{out}}}} - {T_{{\text{set1}}}}}}{{{T_{{\text{out}}}} - {T_{{\text{comf}}}}}}$$

57 $$p_{{{\text{con}}}}^{{{\text{cha}}}}=\frac{{P_{{{\text{ac}}}}^{{{\text{max}}}}\Delta t_{{\text{1}}}^{{{\text{cha}}}}+P_{{{\text{ac}}}}^{{{\text{min}}}}\Delta t_{{\text{2}}}^{{{\text{cha}}}}+{P_{{\text{ac}}1}}(\Delta t - \Delta t_{{\text{1}}}^{{{\text{cha}}}} - \Delta t_{{\text{2}}}^{{{\text{cha}}}})}}{{\Delta t}} - {P_{{\text{ac0}}}}$$

58 $$\Delta t_{1}^{{{\text{dis}}}}={R_{{\text{eq}}}}{C_{{\text{eq}}}}{\text{ln}}\frac{{{T_{{\text{set2}}}} - {T_{{\text{out}}}}+\lambda {P_{\hbox{max} }}{R_{{\text{eq}}}}}}{{{T_{{\text{comf}}}} - {T_{{\text{out}}}}+\lambda {P_{\hbox{max} }}{R_{{\text{eq}}}}}}$$

59 $$\Delta t_{2}^{{{\text{dis}}}}={R_{{\text{eq}}}}{C_{{\text{eq}}}}{\text{ln}}\frac{{{T_{{\text{out}}}} - {T_{{\text{comf}}}}}}{{{T_{{\text{out}}}} - {T_{{\text{set2}}}}}}$$

60 $$p_{{{\text{con}}}}^{{{\text{dis}}}}={P_{{\text{ac0}}}} - \frac{{P_{{{\text{ac}}}}^{{{\text{max}}}}\Delta t_{{\text{1}}}^{{{\text{dis}}}}+P_{{{\text{ac}}}}^{{{\text{min}}}}\Delta t_{{\text{2}}}^{{{\text{dis}}}}+{P_{{\text{ac}}2}}(\Delta t - \Delta t_{{\text{1}}}^{{{\text{dis}}}} - \Delta t_{{\text{2}}}^{{{\text{dis}}}})}}{{\Delta t}}$$

In this study, the most comfortable indoor temperature is selected to be $T_{{comf}} = 26\,^\circ C$, with the upper limit of indoor temperature fluctuation being $T_{{set1}} = 27.5\,^\circ C$, and the lower limit being $T_{{set2}} = 24.5\,^\circ C$.

#### Objective function of real time stage

By integrating hydrogen energy storage for intra-day rescheduling auxiliary services, the discrepancy between the actual interaction power of the integrated energy system and the declared quantity has been essentially compensated for. However, in the real-time stage, rolling optimization is conducted with a time step of half an hour. After obtaining real-time wind and solar data with a precision of half an hour, the air conditioning virtual energy storage, decoupled in time, is utilized to offset the deviations in real-time photovoltaic and wind power generation. This approach enables the actual interaction power between the integrated energy system and the upper-grid to more closely align with the declared power. The objective function for the integrated energy system during the real-time stage is as follows:

61 $$\hbox{min} \sum\limits_{{t=1}}^{{48}} {(C_{t}^{{{\text{pu}}}}+C_{t}^{{{\text{ac}}}})}$$

where ${\text{C}}_{t}^{{{\text{pu}}}}$ denotes the penalty cost associated with the deviation of the declared energy output during the real-time stage; ${\text{C}}_{t}^{{{\text{ac}}}}$ represents the penalty cost incurred by the air conditioning cluster due to adjustments in temperature setpoints. The specific expressions for these costs are delineated as follows:

62 $$C_{t}^{{{\text{pu}}}}=c_{t}^{{{\text{s}}+}}\Delta P_{t}^{{s+}}+c_{t}^{{s - }}\Delta P_{t}^{{{\text{s-}}}}$$

63 $${\text{C}}_{t}^{{{\text{ac}}}}=R_{t}^{{{\text{ac}}}}{\kern 1pt} {\kern 1pt} \left| {{P_{{\text{con}}}}} \right|$$

where $R_{t}^{{{\text{ac}}}}{\kern 1pt} {\kern 1pt}$ is defined as the penalty cost per unit of power for the air conditioning cluster; $\Delta P_{t}^{{s+}}$ and $\Delta P_{t}^{{{\text{s-}}}}$ signifies the positive deviation and negative deviation of the declared power by the IES. The expressions for $\Delta P_{t}^{{s+}}$ and $\Delta P_{t}^{{{\text{s-}}}}$ are presented in Eqs. (64) and (63)

64 $$\Delta P_{t}^{{{\text{s}}+}}={\text{max}}\left( {P_{t}^{{{\text{qgr}}}} - P_{t}^{{{\text{sgr}}}},0} \right)$$

65 $$\Delta P_{t}^{{s{\text{-}}}}={\text{max}}\left( {P_{t}^{{{\text{sgr}}}} - P_{t}^{{{\text{qgr}}}},0} \right)$$

#### Constraints of real time stage

In the real-time optimization stage, the IES is subject to a suite of constraints that ensure not only the normal operation of the system but also its stability and compliance with operational standards. In addition to the power balance constraints, grid interconnection constraints, demand side constraints, and the operational constraints of individual system components, the IES must also adhere to the following constraints:

66 $$- P_{{{\text{con}}}}^{{{\text{cha}}}} \leqslant P_{t}^{{{\text{con}}}} \leqslant P_{{{\text{con}}}}^{{{\text{dis}}}}$$

67 $$\begin{gathered} P_{t}^{{{\text{gt}}}}+P_{t}^{{{\text{PV,rt}}}}+P_{t}^{{{\text{WT,rt}}}}+P_{t}^{{{\text{edis}}}}+\sum\limits_{{i=1}}^{N} {P_{{{\text{ev,}}i{\text{,}}t}}^{{{\text{disc}}}}} +P_{t}^{{{\text{buy}}}}+P_{t}^{{{\text{H2e}}}}+P_{t}^{{{\text{con}}}} \hfill \\ \begin{array}{*{20}{c}} {\begin{array}{*{20}{c}} {}&{}&{} \end{array}}&{}&{} \end{array}=P_{t}^{{{\text{sell}}}}+\sum\limits_{{i=1}}^{N} {P_{{{\text{ev,}}i,t}}^{{{\text{char}}}}} +P_{t}^{{{\text{echa}}}}+0.8P_{t}^{{{\text{el}}}}+\Delta P_{t}^{{{\text{el}}}}+P_{t}^{{{\text{e2H}}}}+P_{t}^{{{\text{sl}}}} \hfill \\ \end{gathered}$$

where $R_{t}^{{{\text{ac}}}}$ denotes the unit cost unit price of power compensation for air-conditioning clusters. $P_{t}^{{{\text{con}}}}$ denotes air conditioning cluster participation in IES auxiliary service response power. $P_{t}^{{{\text{WT,rt}}}}$ and $P_{t}^{{{\text{PV,rt}}}}$ represent the data for WT and PV power output in the real-time stage, respectively.

## Case study

### Basic settings

To validate the aforementioned model, the integrated energy system under investigation encompasses a range of equipment, including gas turbines, energy storage batteries, hydrogen storage systems, gas boilers, waste heat recovery units, wind power generators, photovoltaic panels, and air conditioning clusters. The pollutants in question are carbon dioxide, carbon monoxide, and sulfur dioxide, with their respective emission intensities, environmental valuations, and penalty metrics derived from reference [28]. The cost of natural gas is stipulated at 247 CNY/MWh, with the grid’s electricity purchase time-of-use tariff delineated as follows: an off-peak rate of 361.9 CNY/MWh between 01:00 and 07:00, and from 23:00 to 24:00; a flat rate of 904.7 CNY/MW from 08:00–10:00 and from 15:00–18:00; and a peak rate of 1652 CNY/MW from 11:00 to 14:00, and from 19:00 to 20:00. The electricity sales price is calculated at 80% of the purchase price.

In the day-ahead stage, the electric vehicle’s battery capacity is specified as 65 kWh, with charging and discharging power thresholds set at 7.5 kW, and charging and discharging efficiency factors are 0.95 and 0.9, respectively. The electric vehicles are categorized into a dendrogram of 10 clusters for centralized scheduling via the NJW spectral clustering algorithm. During the intraday stage, the interconversion among electricity, heat, and gas is capped at not exceeding 5% of the instantaneous load, with the transferable load constituting 20% of the aggregate load, further constrained not to surpass 30% of the load at any given moment. The hydrogen storage facility employs a low-pressure hydrogen storage tank with a capacity of 20 kg, and the upper limits for hydrogen production and power generation capabilities are both set at 0.8 MW.

In the real-time dispatch stage, the air conditioning cluster is composed of 1000 uniform air conditioning units. The study imposes a penalty for discrepancies between the actual interchange power of the integrated energy system with the upper grid and the declared quantity, which is set at 1.5 times the real-time purchase electricity tariff and 0.5 times the real-time sales tariff.

To ascertain the veracity of the model formulated within this research, computational simulations are executed utilizing MATLAB in conjunction with the YALMIP/Gurobi optimization suite, harnessed on a personal computing device equipped with a 3.5 GHz Intel Core i9 processor.

### Analysis of optimal scheduling of electric vehicle auxiliary services in the day-ahead stage

#### Algorithm convergence analysis

**Fig. 4:**
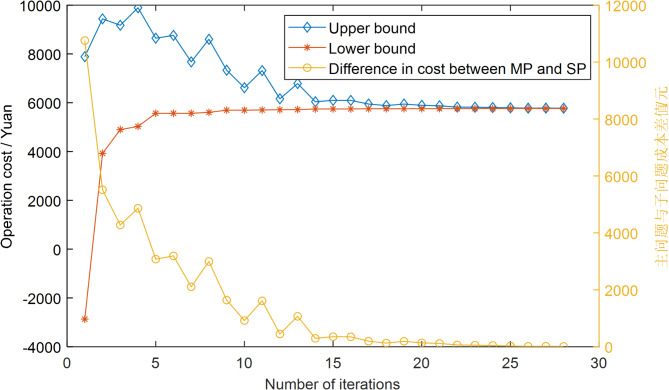
Convergence of C&CG column constraint generation algorithm.

This article employs the Column-and-Constraint Generation method to solve the optimization model, assessing whether the difference between the upper and lower bounds of the objective function values of the master problem and the subproblem meets the convergence accuracy. Figure 4 presents the iterative process of the operational costs of the master and subproblems, as well as the difference between them. It can be observed that the proposed algorithm achieves convergence after 28 iterations, indicating that the C&CG column constraint generation method possesses favorable convergence characteristics.

Furthermore, by examining the operational costs of both the master and subproblems, it is noted that the master problem obtains the optimal operational cost in the first generation (i.e., without considering the penalty costs brought about by the uncertainty of wind and solar power output). The subproblem, starting from the first generation, begins to identify the most adverse scenario for wind and solar power output and gradually compensates for the uncertainty of wind and solar power through the optimization of other decision variables. Based on this, the operational costs of the master and subproblems gradually converge towards consistency until they are equal, which aligns with the core solution philosophy of the C&CG column constraint generation method.

#### Analysis of the optimal scheduling of IES in day-ahead stage

**Fig. 5:**
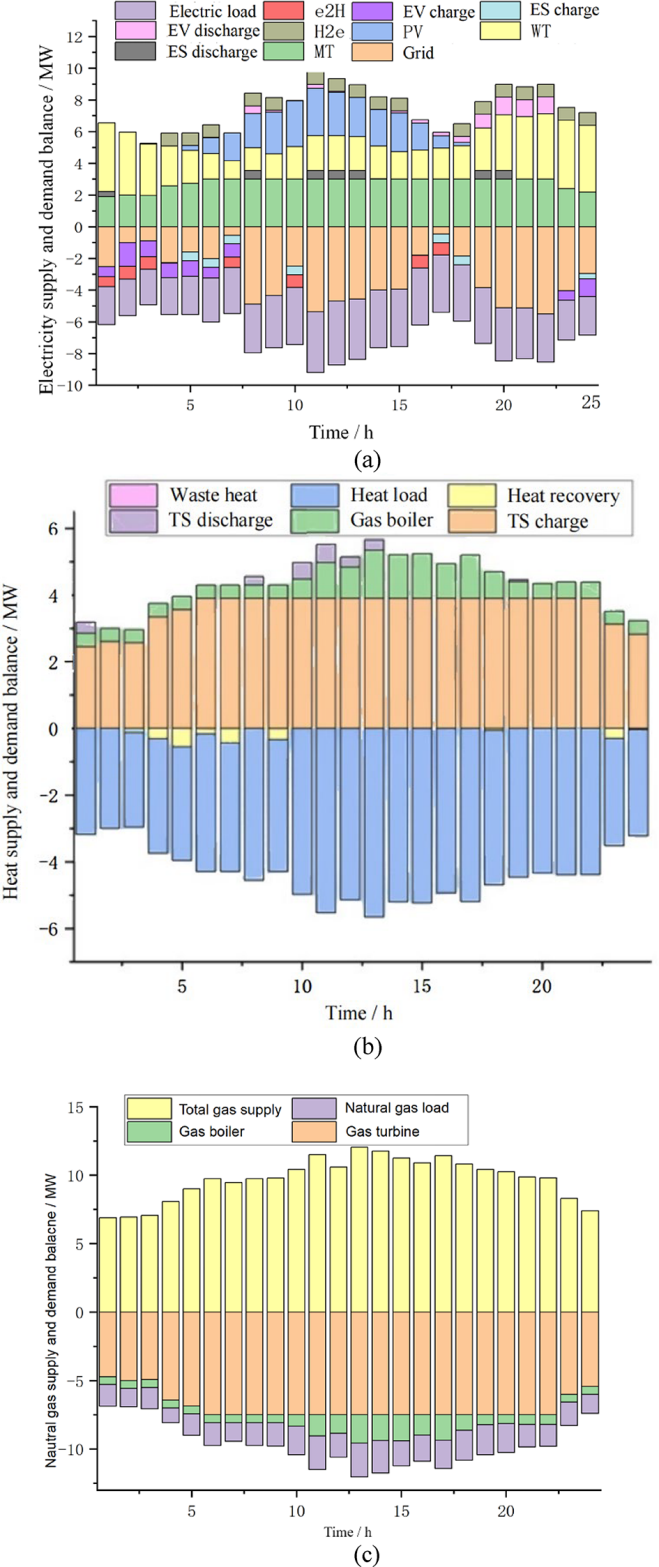
Energy supply/demand balance within the IES. (**a**) Electricity supply/demand balance within IES. (**b**) Thermal supply/demand balance within IES. (**c**) Natural gas supply/demand balance within IES.

Figure 5 provides a comprehensive illustration of the supply and demand balance for electricity, thermal energy, and natural gas in the IES during the day-ahead scheduling stage. Figure 5a presents the optimization strategy for electric vehicles participating in the auxiliary services of the IES during the day-ahead stage. In this study, the storage behaviors of various forms of energy storage are defined such that positive values represent discharging and negative values represent charging. In conjunction with the time-of-use electricity pricing, it can be observed that the IES is primarily powered by gas turbines, photovoltaic, and wind power generation, aligning with the concept of sustainable development. For electric vehicles, during the off-peak electricity pricing periods from 1:00 to 7:00 and 23:00 to 24:00, EVs are charged, with the charging energy provided by gas turbines and wind power. During peak and flat-rate pricing periods, EVs are in a discharging state, with the maximum discharging power occurring during the peak period of 19:00 to 20:00, which maximizes the benefits of the IES. Additionally, by observing the fixed electrical energy storage and hydrogen storage devices, it can be seen that the electrical energy storage devices charge during low electricity pricing periods and discharge during peak pricing periods, adhering to the optimal economic principle of the IES. For the hydrogen storage unit, hydrogen is produced (E2H) and stored during the off-peak electricity pricing periods, and during peak periods, electricity is generated (H2E) through fuel cells to supply the IES. The combination of these three energy storage methods satisfies the “charge low and discharge high” arbitrage behavior and, to some extent, achieves peak shaving and valley filling for the upper grid, alleviating the load pressure on the grid.

Observing Fig. 5b, which illustrates the thermal energy supply and demand balance of the IES, it can be seen that gas turbine power generation meets the electrical load balance of the IES and collects thermal energy through a waste heat recovery system to provide thermal load. During the low thermal load periods, the thermal energy is mainly provided by the waste heat recovery system of the gas turbine, and the thermal storage tank stores the surplus thermal energy during this stage. During peak thermal load periods, the system provides thermal energy through the gas turbine, gas boiler, and thermal storage tank, and in this model, nearly zero heat is wasted, meeting the requirements for green development.

In Fig. 5c, it is observed that the IES primarily consumes natural gas through gas turbines and gas boilers. During peak electricity pricing periods, due to the economic efficiency of gas turbines being superior to the grid electricity price, gas turbines maintain a high level of output to achieve optimal economic benefits. During peak thermal load periods, the model provides thermal energy to the IES by consuming natural gas and collecting waste heat from the gas turbine, resulting in an increase in the consumption of natural gas by the IES during this time period.

### Analysis of the optimal scheduling of hydrogen storage auxiliary services in the intraday stage

**Fig. 6:**
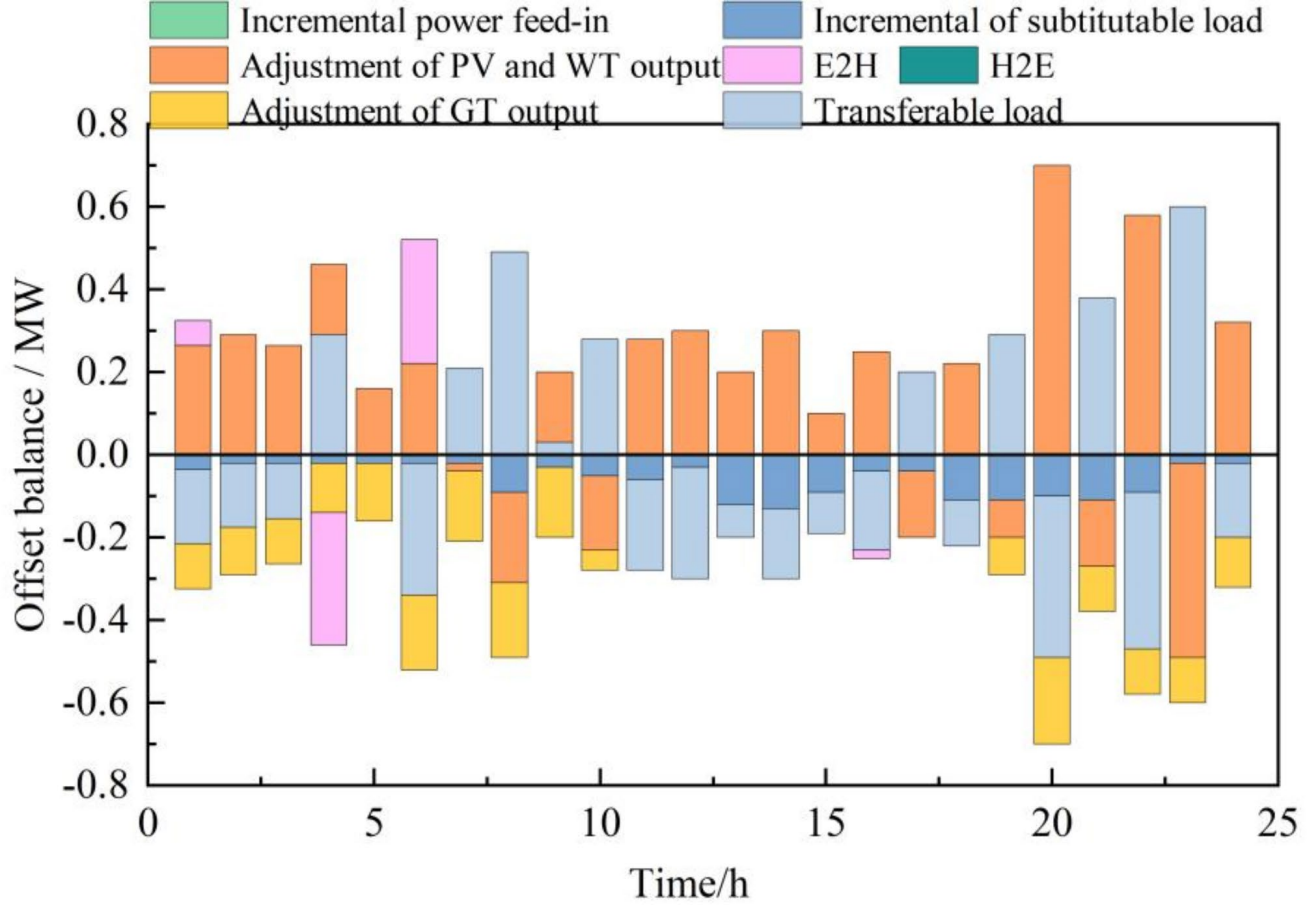
The renewable energy offset operating strategy in intraday stage.

During the intraday stage, more precise data for wind and photovoltaic power generation is obtained on a shorter time scale, which can lead to discrepancies between the actual renewable energy output and the data used in the day-ahead operations. This results in a deviation in the declared power submitted by the IES to the upper grid. Figure 6 presents the optimization strategy for each device to mitigate the wind and photovoltaic deviation, where the deviation is defined as the difference between the intraday output and the day-ahead output. A positive value indicates that the intraday actual output is higher than the day-ahead output, and vice versa. For gas turbines, a positive adjustment indicates that the intraday output is higher than the day-ahead output, and a negative value indicates the opposite.

In the intraday stage, it can be observed that the transferable load, substitutable load, and adjustment of gas turbine output can essentially compensate for the power fluctuations of renewable energy sources. However, there is still a portion of the power that needs to be compensated by the hydrogen storage system: at 1:00 and 6:00, there is a surplus of renewable energy power, mainly absorbed by transferable load, reduction of gas turbine output, and substitutable load, while ensuring the optimal economic operation of the IES by reducing the gas turbine output (negative adjustment of gas turbine power) and also reducing the power-to-hydrogen production during this period. At 5:00, the system has a surplus of renewable energy, and in addition to reducing the gas turbine output and increasing the substitutable load, the hydrogen storage system increases the production to absorb the surplus renewable energy. At 16:00, due to the physical constraints of the gas turbine, it cannot be further adjusted, and the surplus renewable energy power at this moment is absorbed by the transferable load, substitutable load, and increased power-to-hydrogen production of the hydrogen storage system. Therefore, considering the hydrogen storage system can ensure both the economic efficiency of the system and the mitigation of renewable energy output fluctuations.

**Fig. 7:**
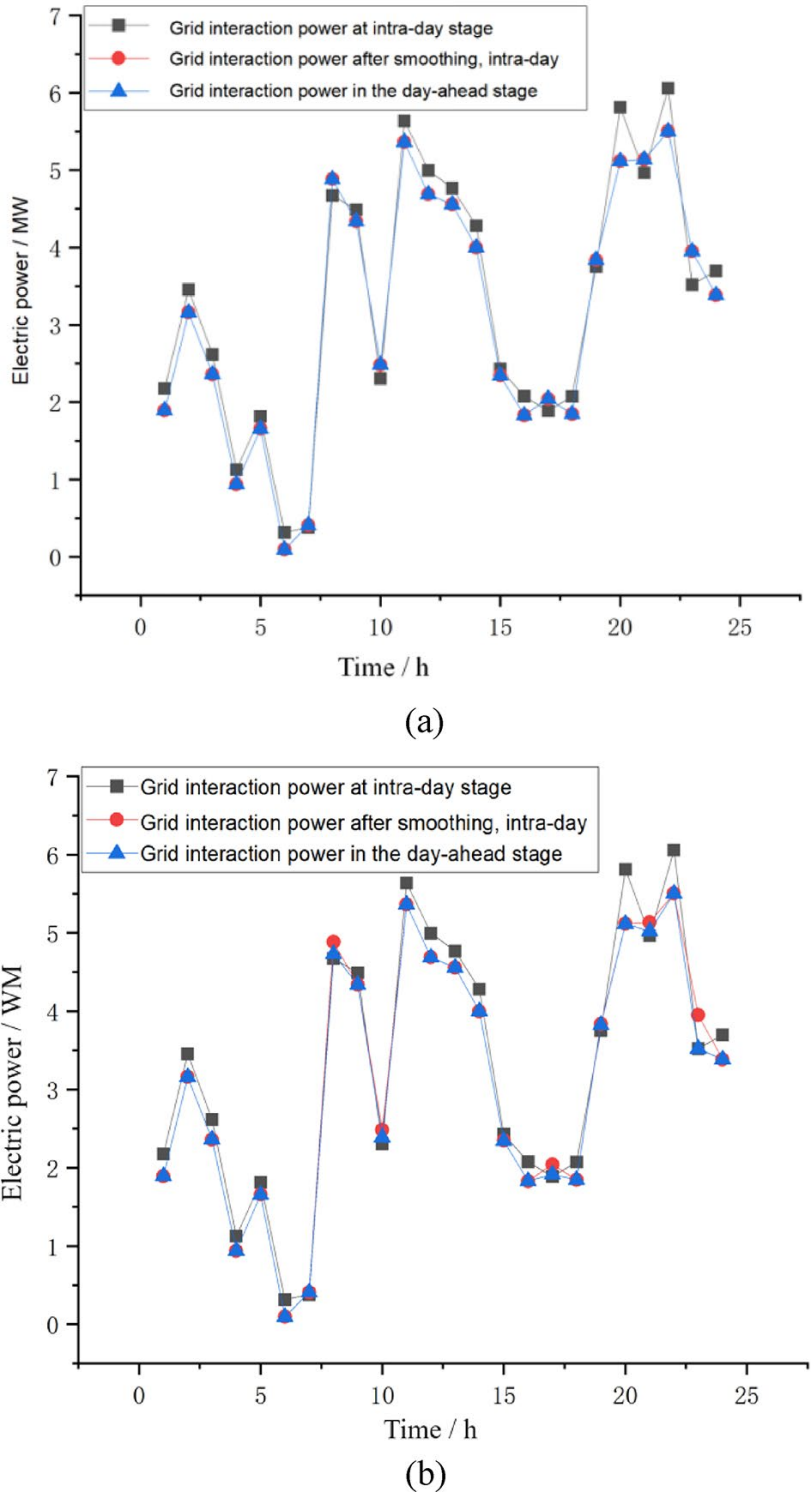
Interactive power scheduling plan between integrated energy system and power grid. (**a**) Considering transferable loads. (**b**) Not considering transferable loads.

Since the day-ahead stage has already determined the IES’s electricity purchase and sale declaration to the grid, the purpose of the optimization operation strategy in the intraday stage is to minimize the deviation caused by the fluctuations in renewable energy output. Figure 6 presents interactive power scheduling plan between integrated energy system and power grid. Comparing Fig. 7a,b, it can be seen that after considering the transferable load, the system has more adjustable load capacity to compensate for the fluctuations in renewable energy power. In Fig. 6a, the interaction power between the IES and the grid is basically completely consistent, showing good mitigation effects. In Fig. 6b, there are certain deviations in the declared quantities between the intraday and day-ahead at 8:00, 10:00, 17:00, 21:00, and 23:00.

### Analysis of optimal scheduling results of virtual energy storage auxiliary services in real-time stage

**Fig. 8:**
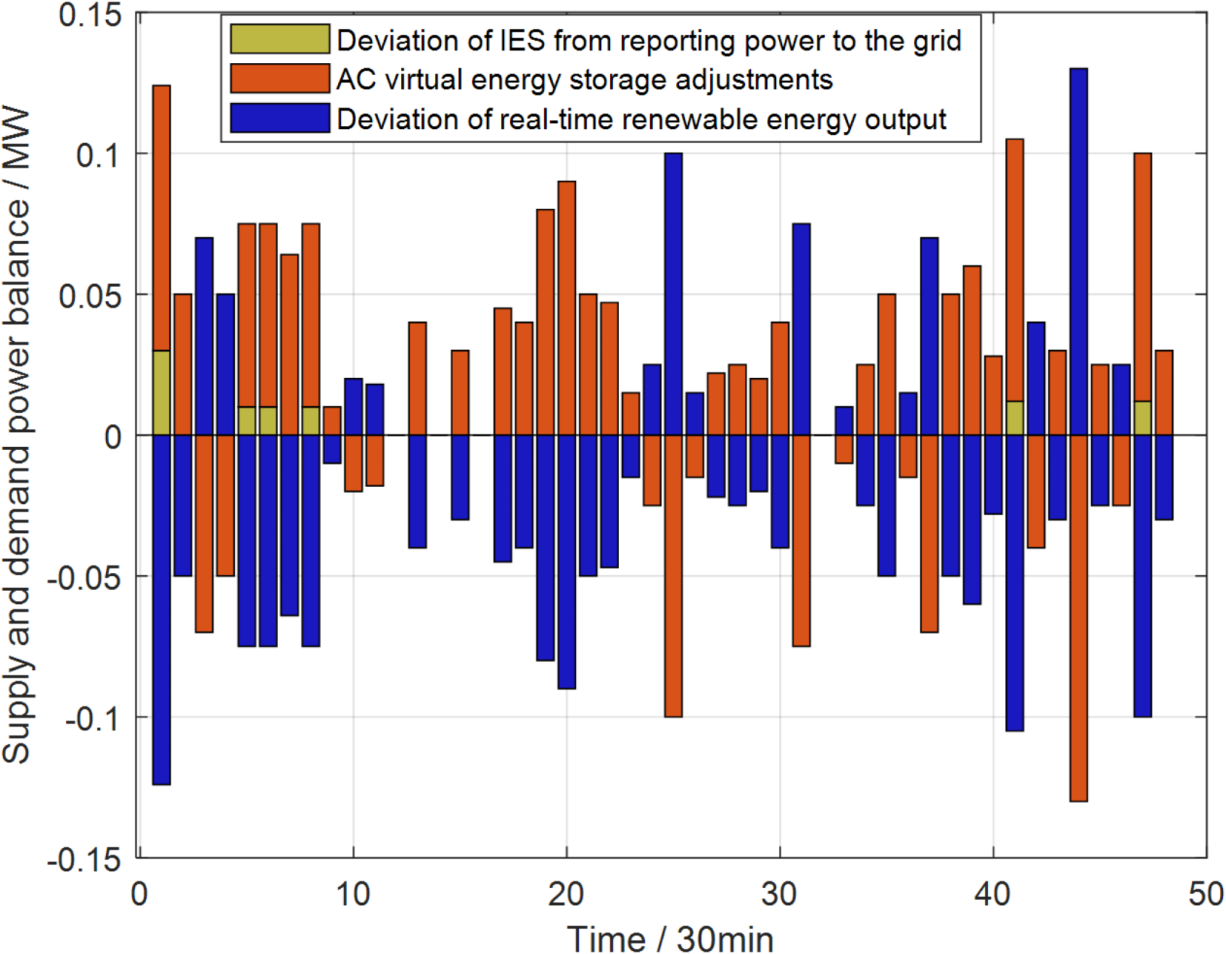
Air conditioning cluster virtual energy storage provides auxiliary services in real-time stage.

In the real-time stage, considering a shorter time scale to obtain precise wind and photovoltaic power generation data, this study employs the air conditioning cluster virtual energy storage, which has the characteristics of energy decoupling and rapid response, to participate in the operation of the IES for auxiliary services. This approach aims to mitigate the power fluctuations caused by renewable energy in the real-time stage. An optimization step length of half an hour is adopted to obtain the real-time operation strategy of the comprehensive air conditioning cluster virtual energy storage, as shown in Fig. 8, where positive values indicate virtual energy storage discharging, and negative values indicate virtual energy storage charging.

Observing Fig. 7, it can be noted that in most moments of the real-time stage, the deviation of renewable energy can be completely compensated by the virtual energy storage of the air conditioning cluster. However, due to the physical constraints of the air conditioning cluster virtual energy storage, at 1:00, 3:00, 3:30, 4:30, 20:30, and 23:30, the deviation of renewable energy cannot be fully compensated. In these instances, the IES must purchase or sell additional electrical energy from or to the upper grid to ensure the lowest operational cost of the IES.

### Comparison of generalized energy storage multi-timescale auxiliary service operation

To validate the effectiveness of the generalized energy storage multi-time scale auxiliary service operation strategy, this study sets up three operational scenarios for comparative analysis:

Scenario 1: Only electric vehicles are considered for participation in the day-ahead auxiliary service optimization operation of the IES.

Scenario 2: Electric vehicles and hydrogen storage are considered for participation in the day-ahead and intraday auxiliary service optimization operations of the IES.

Scenario 3: Electric vehicles, hydrogen storage, and air conditioning cluster virtual energy storage are considered for participation in the day-ahead, intraday, and real-time auxiliary service optimization operations of the IES.

Table 2 presents a detailed breakdown of the operational costs for each equipment in the integrated energy system under the three scenarios mentioned above. As can be seen from Table 1, the operational costs for the integrated energy system are negative in all three scenarios, indicating that the system generates revenue by selling electricity back to the grid. Moreover, the cost of purchasing natural gas accounts for the majority of the total operational costs in each scenario. This is attributed to the fact that the integrated energy system primarily relies on gas turbines and gas-fired boilers to meet the electrical and thermal load demands.

**Table 2:** The operating cost of each equipment of the IES under the three scenarios (CNY).

Scenario	Penalty costs for filing deviations	Cost of purchasing natural gas	Cost of EV reimbursement	Cost of transferable load compensation	Cost of substitutable load penalties	AC reimbursement Costs	Hydrogen storage O&M costs	Power purchase costs	Total operating costs
1	2569.32	57734.70	425.54	0	0	0	1320.6	−63612.53	−2674.39
2	1654.92	54873.08	425.54	864.22	820.71	0	1320.6	−63,613	−2912.39
3	744.51	54873.08	425.54	864.22	820.71	207.56	1320.6	−63566.75	−3568.98

In Scenario 1, the compensation cost for EVs participating in the auxiliary services of the IES is 425.54 CNY, while the penalty cost for the IES due to deviation from the declaration amounts to 2569.32 CNY. Therefore, the day-ahead stage considers the dispatch plan involving EVs in the IES auxiliary services to be economically optimal, and within this plan, the orderly participation of EVs in charging and discharging meets the requirements for the safe and stable operation of the power grid.

In Scenario 2, more accurate wind and solar data are obtained through shorter time scales during the day, resulting in a deviation from the declaration of 1654.92, which is a 35.59% reduction compared to Scenario 1. The operation and maintenance cost of hydrogen energy storage during the day is nearly identical to that of the day-ahead stage, as indicated by the operational strategy in Fig. 5. The overall output power of the hydrogen energy storage remains unchanged, and its operation and maintenance cost remains essentially stable. The costs for purchasing natural gas and pollution penalties are reduced by 2861.7 CNY and 23.5 CNY, respectively. This reduction is due to the consideration of alternative and shiftable loads in Scenario 2, along with a readjusted hydrogen energy storage operation strategy. This approach optimizes the output curve of the gas turbine, aligns with the sustainable development strategy under the dual-carbon context, and effectively mitigates the power fluctuations of renewable energy sources. The electricity purchase cost during the day and the day-ahead stages remains essentially the same. Ultimately, the revenue in the day stage increases by 238.09 CNY compared to the day-ahead operation strategy, meeting the optimal economic operation requirements of the IES.

In Scenario 3, the real-time stage obtains even more precise wind and solar data compared to the mid-day, leading to a deviation from the declaration cost of 744.51 CNY, which is a 55.01% reduction from Scenario 2. In this scenario, air conditioning cluster virtual energy storage, characterized by rapid response and time-decoupling features, is employed to mitigate the power fluctuations of renewable energy sources in real time. Comparing the electricity purchase costs between Scenario 2 and Scenario 3 shows that while Scenario 3 cannot fully compensate for the uncertainty of renewable energy sources, it still provides a significant mitigation effect. The difference in electricity purchase costs is 46.25 CNY, due to the physical constraints of the air conditioning virtual energy storage, which limits the adjustable capacity. At this point, the final operational revenue in Scenario 3 amounts to 3568.98 CNY, which is an increase of 656.59 CNY compared to Scenario 2.

### The impact of virtual energy storage in air conditioning clusters on the real-time stage

As delineated in Sect. 3.6 concerning the real-time stage scheduling strategy for the air conditioning cluster virtual energy storage, the real-time charging and discharging capacity of the air conditioning cluster virtual energy storage dictates the transactional electricity volume between the integrated energy system and the electricity market during the real-time stage. Equation (57) elucidates that by judiciously modifying the range of the indoor optimal temperature, a more substantial virtual energy storage adjustment capacity can be procured, thus more effectively offsetting the real-time fluctuations in renewable energy power generation.

Table 3 enumerates three distinct solution for adjusting the upper and lower bounds of the indoor adjustable temperature. A comparative analysis of the outcomes from these strategies substantiates the capability of the air conditioning cluster virtual energy storage to smooth the real-time oscillations in renewable energy power during the real-time stage.

**Table 3:** Comparison of the results of three temperature operation scenarios for air conditioning clusters.

Solution	Temperature upper limit (℃)	Temperature lower limit (℃)	Optimal temperature (℃)	Total charging capacity (MWh)	Total discharging capacity (MWh)	Penalty cost (CNY)
1	27.5	24.5	26	0.68	1.47	0
2	27.0	24.5	26	0.68	1.40	46.25
3	27.0	24.0	26	0.68	1.40	46.25

**Fig. 9:**
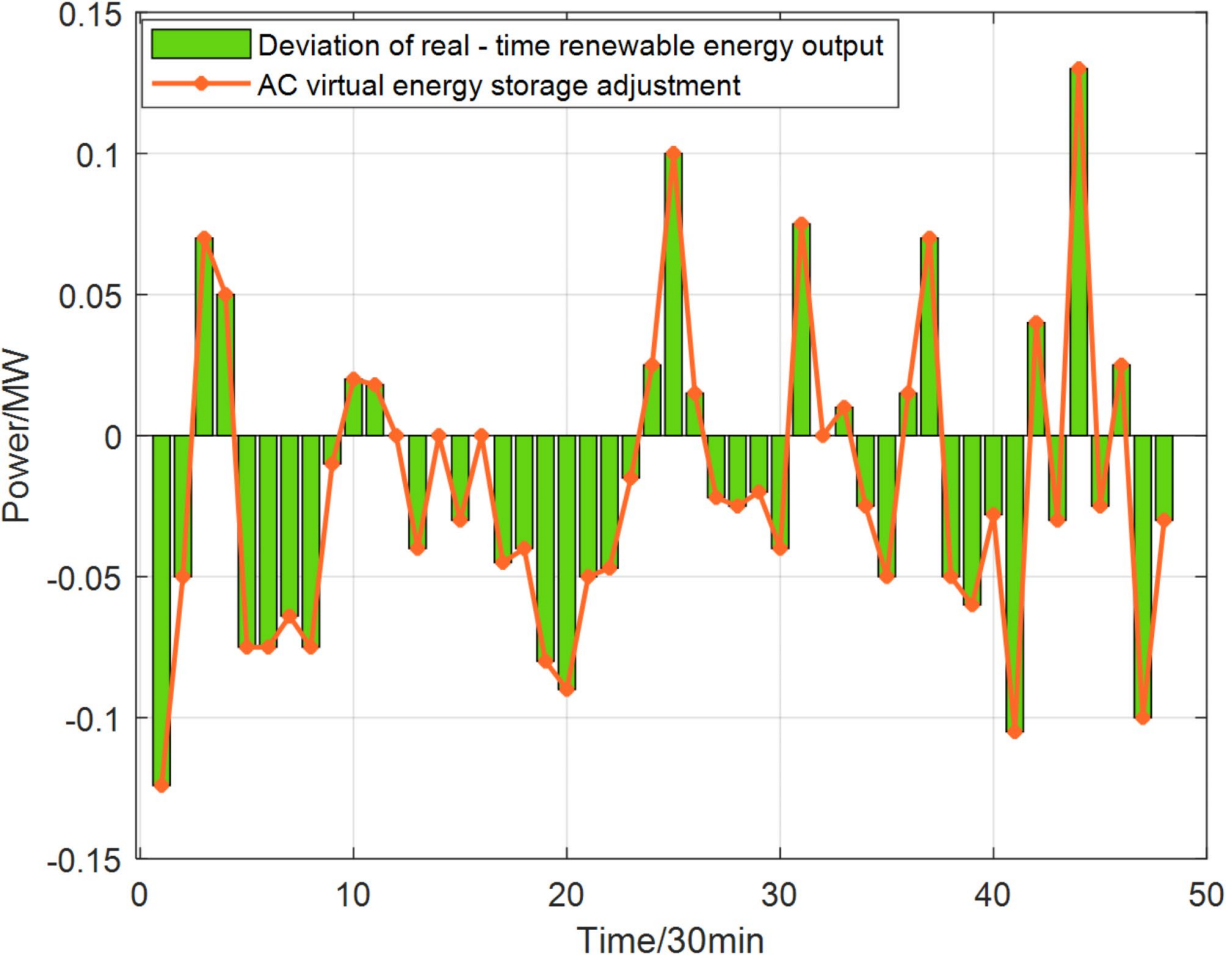
Virtual energy storage for air conditioning clusters operation strategy in Solution 1.

As shown in Table 2, Solution 2 represents the charging and discharging parameters of the virtual energy storage for the air conditioning cluster proposed in this paper. Solution 1 increases the highest adjustable indoor temperature, and the experimental results indicate that the total amount of virtual energy storage charging for the air conditioning cluster remains unchanged. However, the total discharging amount is increased by 0.07 MWh, which is consistent with the simulation results depicted in Fig. 9. The real-time leveling effect of Solution 1 is illustrated in Fig. 9. In contrast, Solution 3 reduces the lowest adjustable indoor temperature, and it can be observed that the total charging and discharging power of the air conditioning cluster’s virtual energy storage remains unchanged. From Table 3, we can conclude that by appropriately adjusting the upper limit of indoor temperature, the power uncertainty of renewable energy sources can be further compensated for.

## Conclusions

The multi-timescale optimization scheduling model for the IES has proven to be a robust framework for enhancing operational efficiency and economic viability. Key outcomes of the study include:

1. Innovative Scheduling Strategy: he integration of EVs, hydrogen storage, and air conditioning clusters across day-ahead, intraday, and real-time stages has demonstrated an adaptive and responsive approach to energy supply and demand variability.
2. Cost Reduction: The optimized scheduling has achieved a notable reduction in operational costs, with Scenario 3 showcasing a 656.59 CNY decrease compared to Scenario 2, highlighting the economic advantages of the proposed strategy.
3. Renewable Energy Fluctuation Mitigation: The real-time stage’s virtual energy storage model has effectively mitigated renewable energy fluctuations, with a 55.01% reduction in deviation costs, as evidenced by a penalty cost of 744.51 CNY in Scenario 3.
4. Virtual Energy Storage Optimization: By appropriately adjusting the upper limit of indoor temperature, the power uncertainty of renewable energy sources can be further compensated for.

Future research in IESs should focus on refining the practical integration of hydrogen and battery energy storage, as well as the optimal deployment of electric vehicles, by considering their capacities and the impact on system performance. It will also be crucial to address the complexities of stakeholder interests, especially when these energy components are managed by different entities with potentially conflicting objectives.

## Electronic supplementary material

Below is the link to the electronic supplementary material.

Supplementary Material 1

## Data Availability

Data is provided within the manuscript or supplementary information files.

## References

[1] Wang, Y. et al. State-of-the-art review on evaluation indicators of integrated intelligent energy from different perspectives. *Renew. Sustain. Energy Rev.* **189**, 113835. 10.1016/j.rser.2023.113835 (2024).

[2] Zhang, S. et al. An overview of reinforcement learning-based approaches for smart home energy management systems with energy storages. *Renew. Sustain. Energy Rev.* **185**, 113606. 10.1016/j.rser.2023.113606 (2023).

[3] Chen, M., Lu, H., Chang, X. & Liao, H. An optimization on an integrated energy system of combined heat and power, carbon capture system and power to gas by considering flexible load. *Energy* **273**, 127203 (2023).

[4] Fu, Y. et al. Optimal configuration method of demand-side flexible resources for enhancing renewable energy integration. *Sci. Rep.* **14**, 7658. 10.1038/s41598-024-58266-6 (2024). 38561382 10.1038/s41598-024-58266-6 PMC10985078

[5] Ma, H. et al. An effective planning approach for integrated energy systems considering equipment operating characteristics. *Heliyon* **9** (11), e21409 (2023). 38028009 10.1016/j.heliyon.2023.e21409 PMC10651462

[6] Elshurafa, A. M. The value of storage in electricity generation: A qualitative and quantitative review. *J. Energy Storage* **32**, 101872. 10.1016/j.est.2020.101872 (2020).

[7] Hou, M., Zhao, Y. & Ge, X. Optimal scheduling of the plug-in electric vehicles aggregator energy and regulation services based on grid to vehicle. *Int. Trans. Electr. Energy Syst.* **27**, e2364. 10.1002/etep.2364 (2017).

[8] Li, W. et al. Two stage stochastic energy scheduling for multi energy rural microgrids with irrigation systems and biomass fermentation. *IEEE Trans. Smart Grid Early Access*10.1109/TSG.2024.3483444 (2024).

[9] Yang, Z. et al. A multi-stage stochastic dispatching method for electricity-hydrogen integrated energy systems driven by model and data. *Appl. Energy* **371**, 123668 (2024).

[10] Mao, Y. et al. A collaborative demand-controlled operation strategy for a multi-energy system. *IEEE Access* **9**, 80571–80581. 10.1109/ACCESS.2021.3083922 (2021).

[11] Jiang, M. et al. Integrated demand response modeling and optimization technologies supporting energy internet. *Renew. Sustain. Energy Rev.* **203**, 114757 (2024).

[12] Song, Y. et al. Optimal scheduling of zero-carbon integrated energy system considering long- and short-term energy storages, demand response, and uncertainty. *J. Clean. Prod.* **435**, 140393 (2024).

[13] Mohseni, S., Alan, C. B., Kelly, S. & Will, N. B. Demand response-integrated investment and operational planning of renewable and sustainable energy systems considering forecast uncertainties: A systematic review. *Renew. Sustain. Energy Rev.* **158**, 112095 (2022).

[14] Liana, T. et al. Demand response optimization for smart grid integrated buildings: review of technology enablers landscape and innovation challenges. *Energy Build.* **326**, 115067 (2025).

[15] Basu, M. Optimal generation scheduling of hydrothermal system with demand side management considering uncertainty and outage of renewable energy sources. *Renew. Energy* **146**, 530–542. 10.1016/j.renene.2019.06.069 (2020).

[16] Jiao, X. et al. An optimal method of energy management for regional energy system with a shared energy storage. *Energies* **16** (2), 886. 10.3390/en16020886 (2023).

[17] Chen, C. et al. Coordinated siting and sizing for integrated energy system considering generalized energy storage. *Int. J. Electr. Power Energy Syst.* **155**, 109619. 10.1016/j.ijepes.2023.109619 (2024).

[18] Luo, L. et al. Coordinated scheduling of generalized energy storage in multi-voltage level AC/DC hybrid distribution network. *J. Energy Storage* **57**, 106189. 10.1016/j.est.2022.106189 (2023).

[19] He, S. et al. Worst CVaR based energy management for generalized energy storage enabled building-integrated energy systems. *Renew. Energy* **203**, 255–266106750 10.1016/j.renene.2022.12.017.

[20] Feng, Z. et al. A production and transport scheduling strategy of energy and resources of pelagic clustering Islands based on generalized movable energy storage. *Energy Rep.* **11**, 5654–5667. 10.1016/j.egyr.2024.05.034 (2024).

[21] Xia, Y., Xu, Q., Qian, H., Liu, W. & Sun, C. Bilevel optimal configuration of generalized energy storage considering power consumption right transaction. *Int. J. Electr. Power Energy Syst.* **128**, 106750. 10.1016/j.ijepes.2020.106750 (2021).

[22] Montoya, O., Garcés, A. & Espinosa-Pérez, G. A generalized passivity-based control approach for power compensation in distribution systems using electrical energy storage systems. *J. Energy Storage* **16**, 259–268. 10.1016/j.est.2018.01.018 (2018).

[23] Zhao, J., Zhang, H. & Wang, C. Distributed state-of-charge and power balance estimation for aggregated battery energy storage systems with EV aggregators. *Energy* **305**, 132193. 10.1016/j.energy.2024.132193 (2024).

[24] Khan, M., Rehman, T., Hussain, A. & Kim, H. Day-ahead operation of a multi-energy microgrid community with shared hybrid energy storage and EV integration. *J. Energy Storage* **97**, 112855. 10.1016/j.est.2024.112855 (2024).

[25] Kumar, V., Naresh, R. & Sharma, V. Stochastic profit-based unit commitment problem considering renewable energy sources with battery storage systems and plug-in hybrid electric vehicles. *Int. J. Energy Res.* **46**, 16445–16460 (2022).

[26] National Household Travel Survey. http://nhts.ornl.gov/ [Accessed: 1 February 2022].

[27] Thakur, D., Kumar, S., Kumar, V. & Kaur, T. Estimation of calorific value using an artificial neural network based on stochastic ultimate analysis. *Renew. Energy* **228**, 120668. 10.1016/j.renene.2024.120668 (2024).

