# Supplementary material for: Multi-timescale optimization scheduling of integrated energy systems oriented towards generalized energy storage services
Source: Sci Rep. 2025 Mar 12;15:8549. doi: 10.1038/s41598-025-92601-9 (PMC11903860; doi:10.1038/s41598-025-92601-9)
Supplement: Supplementary file 1 — Supplementary Material 1 [file 41598_2025_92601_MOESM1_ESM.docx]

**APPENDIX A**

**Table A1.** Pollutant emission of gas turbine

| Pollutant emission | NO*_x_* | CO_2_ | CO | SO_2_ |
| --- | --- | --- | --- | --- |
| Emissions (kg/MWh) | 0.6818 | 184.0829 | 0.1702 | 0.000928 |
| Environmental value (Yuan/kg) | 7.00 | 0.020125 | 0.875 | 5.25 |
| Penalty price (Yuan/kg) | 1.75 | 0.00875 | 0.14 | 0.875 |


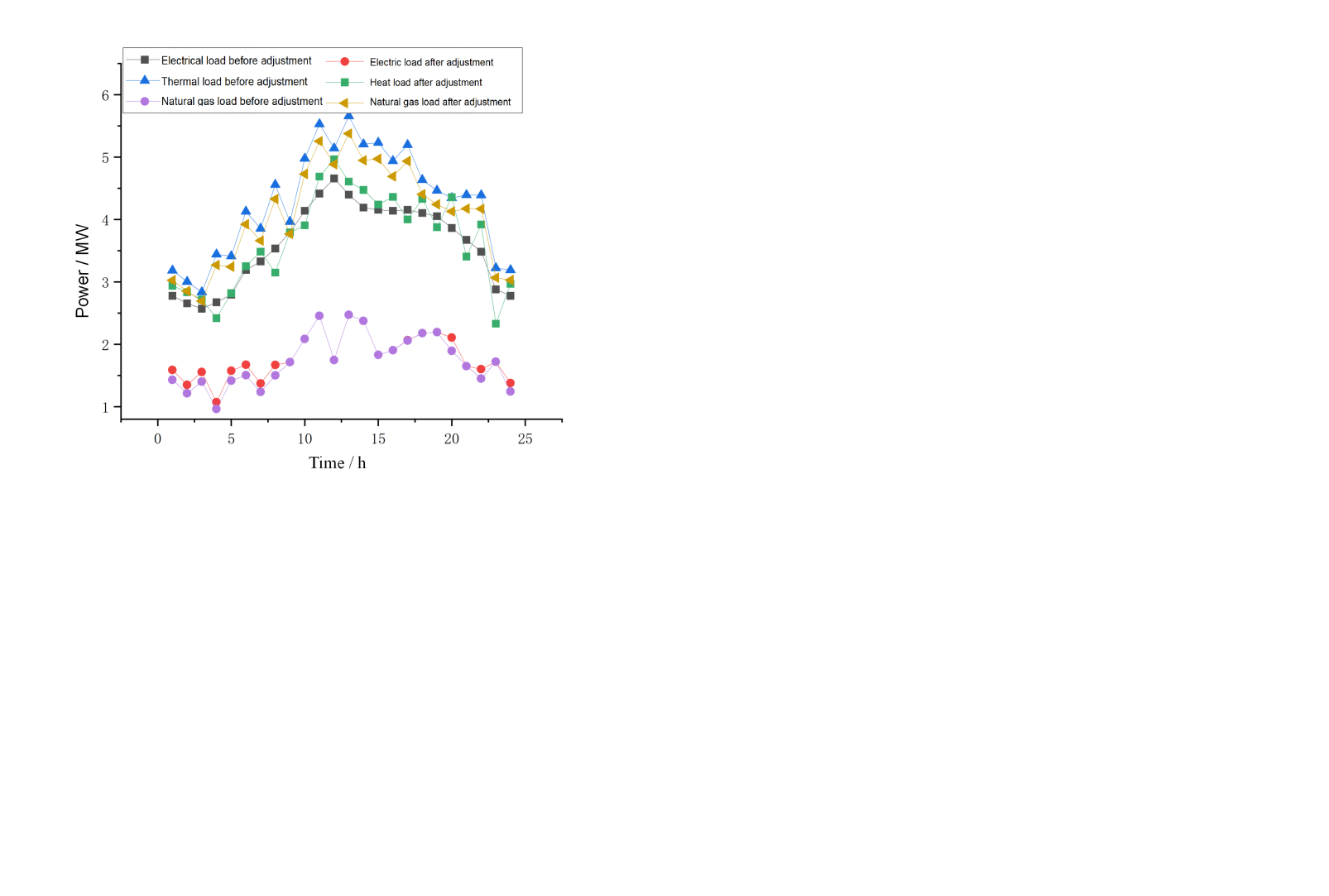


Figure A1 The various types of loads before and after implementing demand response


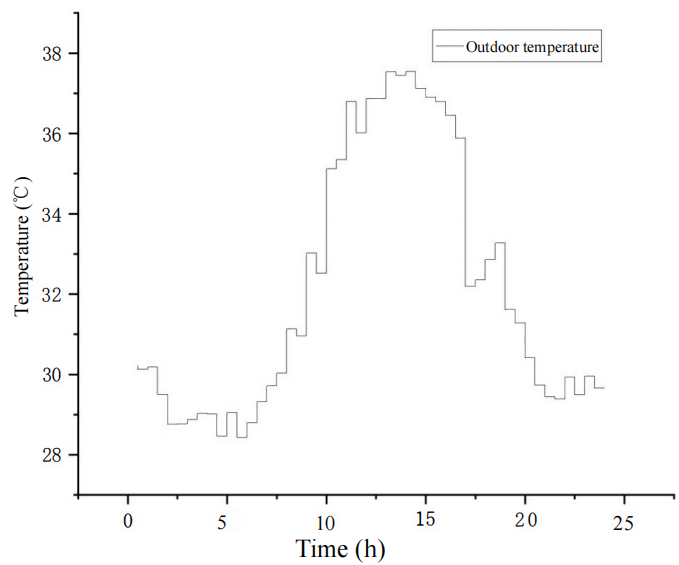


Figure A2 Outdoor temperature


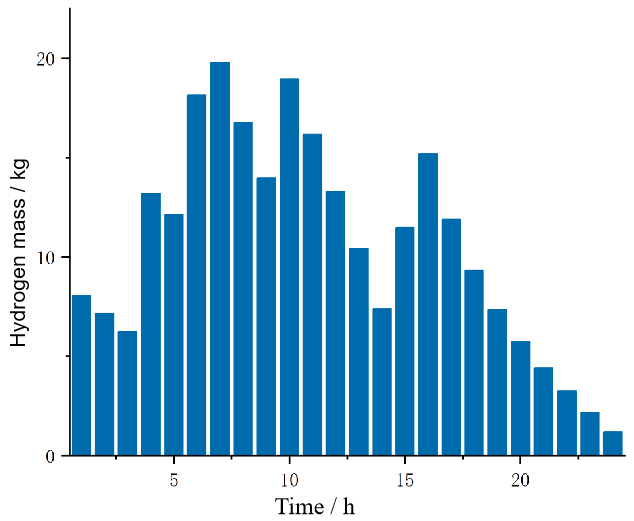


Figure A3 The charge of state of hydrogen storage tank


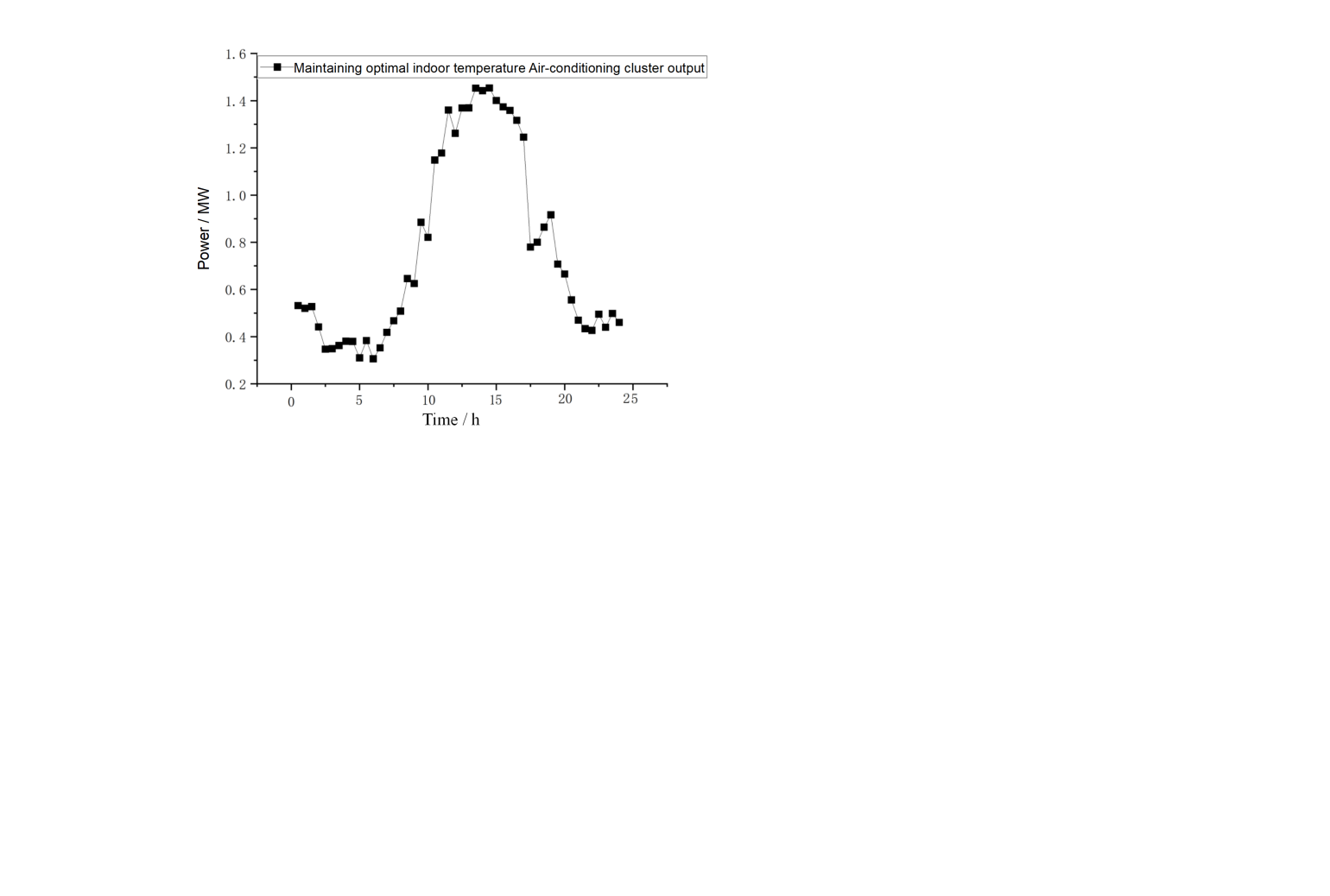


Figure A4 The power of the air-conditioning cluster to maintain the most comfortable temperature


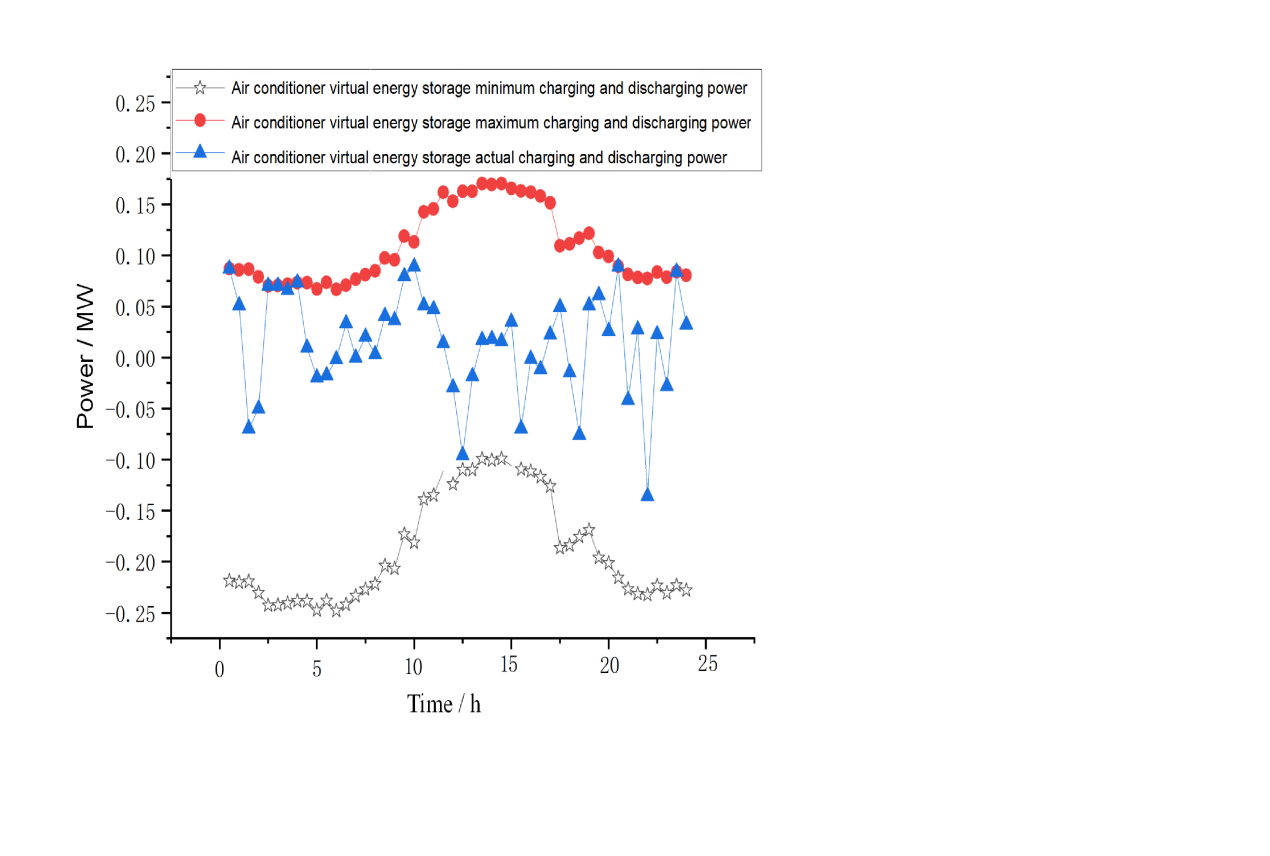


Figure A5 Real-time power of air conditioning virtual energy storage.

**APPENDIX B**

The departure and arrival times of electric vehicles, as well as the number of trips, are described by the following probability density functions:

$f_{\text{dep}}(x)=\left\{\begin{matrix} \frac{1}{\sqrt{2\pi}\sigma_{\text{dep}}}\exp\left[-\frac{\left(x-\mu_{\text{dep}}\right)^{2}}{2\sigma_{_{\text{dep}}}^{2}}\right]\text{，}\begin{matrix} & \end{matrix}0<x\leq(\mu_{\text{dep}}+12) \\ \frac{1}{\sqrt{2\pi}\sigma_{\text{dep}}}\exp\left[-\frac{\left(x-24-\mu_{\text{dep}}\right)^{2}}{2\sigma_{_{\text{dep}}}^{2}}\right],\begin{matrix} & \end{matrix}(\mu_{\text{dep}}+12)<x\leq24\begin{matrix} \\ \end{matrix} \end{matrix}\right.$ (B1)

$f_{\text{arr}}(x)=\left\{\begin{matrix} \frac{1}{\sqrt{2\pi}\sigma_{\text{arr}}}\exp\left[-\frac{\left(x+24-\mu_{\text{arr}}\right)^{2}}{2\sigma_{\text{arr}}^{2}}\right]\text{，}\begin{matrix} & \end{matrix}0<x\leq(\mu_{\text{arr}}-12) \\ \frac{1}{\sqrt{2\pi}\sigma_{\text{arr}}}\exp\left[-\frac{\left(x-\mu_{\text{arr}}\right)^{2}}{2\sigma_{_{\text{arr}}}^{2}}\right]\text{，}\begin{matrix} & \end{matrix}(\mu_{\text{arr}}-12)<x\leq24 \end{matrix}\right.$ (B2)

$L_{\text{c}}(x)=\frac{1}{x\sigma_{\text{c}}\sqrt{2\pi}}\exp\left[-\frac{\left(\text{In}x-\mu_{\text{c}}\right)^{2}}{2\sigma_{\text{c}}^{2}}\right]$ (B3)

where $f_{\text{dep}}(x)$ and $f_{\text{arr}}(x)$ represent departure and arrival times of electric vehicles probability density functions, respectively; $\mu_{\text{dep}}$ $\mu_{\text{arr}}$ and $\mu_{\text{c}}$ represent the expected values for the disconnection time, connection time, and the number of trips, respectively, with values of 8.92, 17.47, and 3.98; $\sigma_{\text{dep}}$, $\sigma_{\text{arr}}$, $\sigma_{\text{c}}$ represent the standard deviations for the disconnection time, connection time, and the number of trips, respectively, with values of 3.24, 3.41, and 1.14.

After employing Latin hypercube sampling, this study utilizes NJW spectral clustering to categorize 200 electric vehicles into 10 clusters. The disconnection and reconnection times, as well as the state of charge upon conclusion for the electric vehicle clusters, are presented in Table B1.

**Table B1.**  The characteristics of electric vehicle after clustering

| Cluster Category | Grid Connection Time | Grid Disconnection Time | SOC at Grid Connection |
| --- | --- | --- | --- |
| 1 | 14:15 | 7:53 | 0.826 |
| 2 | 16:54 | 5:18 | 0.858 |
| 3 | 21:59 | 11:35 | 0.789 |
| 4 | 18:45 | 9:19 | 0.515 |
| 5 | 18:03 | 10:40 | 0.863 |
| 6 | 17:48 | 7:42 | 0.759 |
| 7 | 13:45 | 2:54 | 0.819 |
| 8 | 17:51 | 7:57 | 0.858 |
| 9 | 18:47 | 3:02 | 0.854 |
| 10 | 20:54 | 7:13 | 0.836 |

**Figure legends**

**Figure A2** Outdoor temperature

**Figure A3** The charge of state of hydrogen storage tank

**Figure A4** The power of the air-conditioning cluster to maintain the most comfortable temperature

**Figure A5** Real-time power of air conditioning virtual energy storage
